# Hedycaryol – Central Intermediates in Sesquiterpene Biosynthesis, Part II

**DOI:** 10.1002/chem.202200405

**Published:** 2022-03-21

**Authors:** Houchao Xu, Jeroen S. Dickschat

**Affiliations:** ^1^ Kekulé-Institute of Organic Chemistry and Biochemistry University of Bonn Gerhard-Domagk-Straße 1 53121 Bonn Germany

**Keywords:** biosynthesis, configuration determination, hedycaryol, sesquiterpenes, structure elucidation

## Abstract

The known sesquiterpenes that arise biosynthetically from hedycaryol are summarised. Reasonings for the assignments of their absolute configurations are discussed. The analysis provided here suggests that reprotonations at the C1=C10 double bond of hedycaryol are directed toward C1 and generally lead to 6–6 bicyclic compounds, while reprotonations at the C4=C5 double bond occur at C4 and result in 5–7 bicyclic compounds. Read more in the Review by H. Xu and J. S. Dickschat (DOI: 10.1002/chem.202200405).

## Graphical abstract

**Hedycaryol is an** important intermediate in the biosynthesis of sesquiterpenes. This review discusses the unique chemistry of hedycaryol, its occurrence in Nature and all known compounds derived from it by terpene cyclisations. Structure elucidations including structural revisions and reasoning for the assignment of absolute configurations of the discussed compounds are presented.

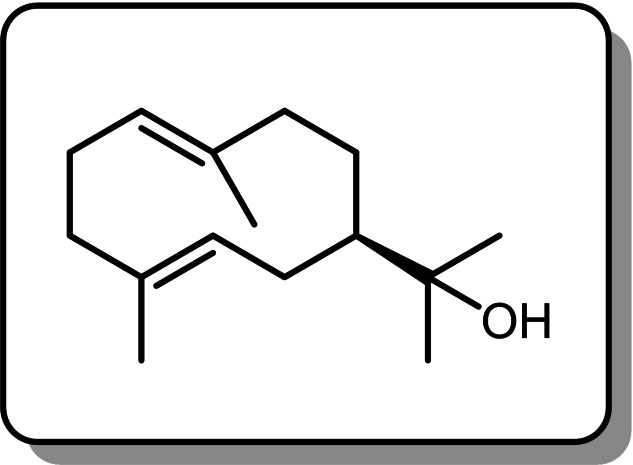

## 1 Introduction

**Scheme 1:**
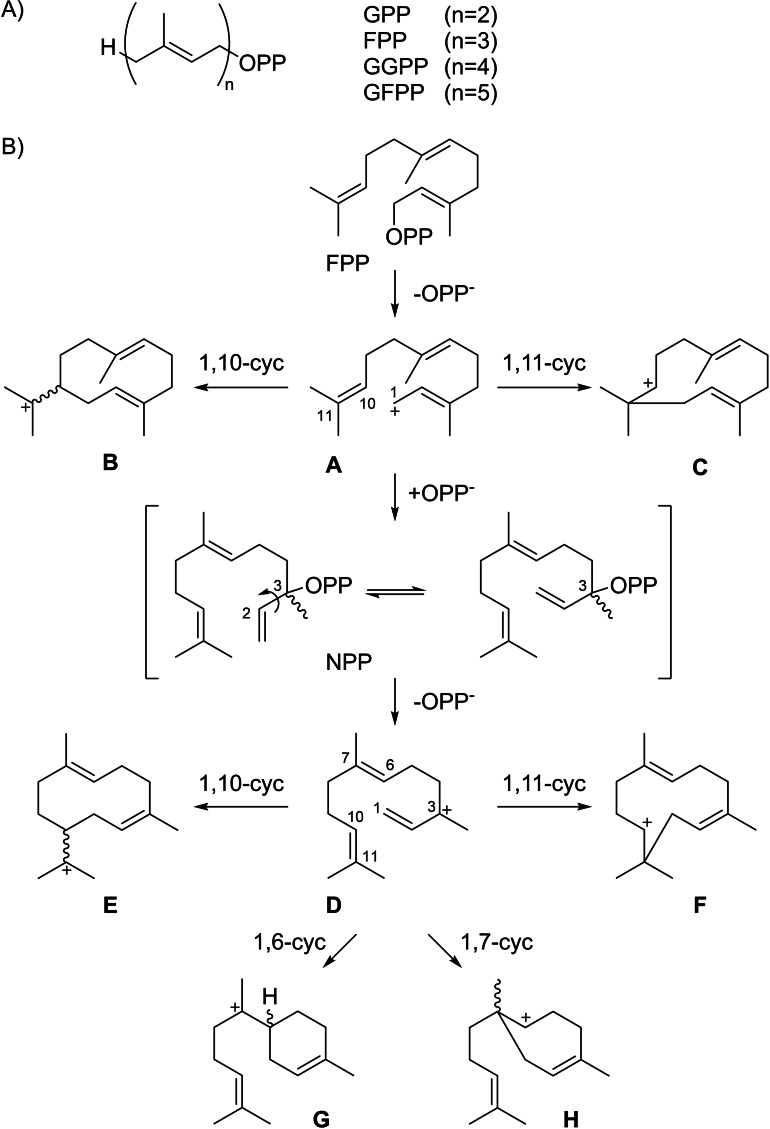
Terpene biosynthesis. A) Structures of oligoprenyl diphosphates. B) Cyclisation modes of FPP towards sesquiterpenes.

Terpenoids represent the largest class of natural products, exhibit an extraordinary structural diversity and complexity, and are often associated with remarkable biological and pharmaceutical activities.^[1]^ Their carbon skeletons are assembled through the action of terpene synthases from only a few acyclic precursors, oligoprenyl diphosphates, that contain multiples of five carbon units with an alkene function and a methyl branch and follow the general formula H‐(C_5_H_8_)_n_‐OPP (Scheme 1A). During the past decades, many type I terpene synthases have been characterised from plants,[^2^, ^3^, ^4^] bacteria,[^4^, ^5^] fungi[^4^, ^6^] and protists^[7]^ that act on their substrates through diphosphate abstraction, followed by a cationic cascade reaction to yield usually (poly)cyclic terpene hydrocarbons or alcohols. Subclasses of these enzymes include monoterpene synthases for the conversion of geranyl diphosphate (GPP, C_10_, n=2) and sesquiterpene synthases that act on farnesyl diphosphate (FPP, C_15_, n=3). For diterpene and sesterterpene synthases[^4^, ^8^] the substrates geranylgeranyl diphosphate (GGPP, C_20_, n=4) and geranyl farnesyl diphosphate (GFPP, C_25_, n=5) with their multiple reactive double bonds allow for highly complex cyclisation cascades, leading to a fascinating structural complexity from a simple acyclic molecule in just one enzymatic step. Site‐directed mutagenesis experiments gave detailed insights into terpene synthase catalysis and made enzymes with new functions available,^[9]^ and also the conversion of non‐natural substrate analogues is possible,^[10]^ making terpene synthases particularly interesting for the enzymatic synthesis of molecules with highly complex architectures. Finally, heterologous expression approaches in engineered yeast^[11]^ or *Escherichia coli* strains^[12]^ add to the successful methodical repertoire of modern terpene synthase applications.

Type I terpene synthases ionise oligoprenyl diphosphates through the abstraction of diphosphate to yield a highly reactive allyl cation that can subsequently undergo a cascade reaction composed of several elementary steps including cyclisation reactions by intramolecular attack of an alkene function to a cationic centre, Wagner‐Meerwein rearrangements, hydride or proton shifts, and a final deprotonation or capture with water. In some cases the deprotonation to an electrically neutral compound is followed by a reprotonation event to initiate a second cyclisation cascade. Herein, for the deprotonation‐reprotonation sequence combined experimental and theoretical studies have revealed the importance of main chain carbonyl oxygens and an active site water for the bacterial selinadiene synthase.[^13^, ^14^]

For the conversion of FPP by sesquiterpene synthases different initial cyclisation events are possible (Scheme 1B).[^15^, ^16^] After ionisation of FPP to the farnesyl cation (**A**), a 1,10‐cyclisation can lead to the (*E*,*E*)‐germacradienyl cation (**B**) or a 1,11‐cyclisation may result in the (*E*,*E*)‐humulyl cation (**C**). Alternatively, the abstracted diphosphate can re‐attack at C3 to give nerolidyl diphosphate (NPP) that can undergo a conformational change through rotation around its C2‐C3 single bond. Its reionisation to **D** opens four more cyclisation options through 1,10‐cyclisation to the (*Z*,*E*)‐germacradienyl cation (**E**), 1,11‐cyclisation to the (*Z*,*E*)‐humulyl cation (**F**), 1,6‐cyclisation to the bisabolyl cation (**G**) and 1,7‐cyclisation to **H**. For all chiral intermediates both enantiomers can be reached through these processes.

Intermediate **B** can be deprotonated to yield germacrene A that is a widespread intermediate towards many eudesmane and guaiane sesquiterpene hydrocarbons that can be formed through its reprotonation‐induced transannular reactions. The accumulated knowledge about this class of sesquiterpenes was recently summarised by us in a review article in this journal.^[17]^ We have also performed a computational study to explore the chemical space through downstream hydride shifts for the different stereoisomers of the guaianes, showing that (suprafacial) 1,2‐hydride shifts are always possible, while 1,3‐hydride migrations can only be realised for certain geometries of the guaiane skeletons.^[18]^ As an alternative to the deprotonation to germacrene A, cation **B** can also be captured by water to yield the sesquiterpene alcohol hedycaryol, which is a likewise important intermediate toward many sesquiterpene alcohols. Here we provide a comprehensive overview of the chemistry of hedycaryol and the compounds derived from it through terpene cyclase mediated downstream cyclisations.

## 2 Hedycaryol

### 2.1 Structure elucidation and occurrence in Nature

**Scheme 2:**
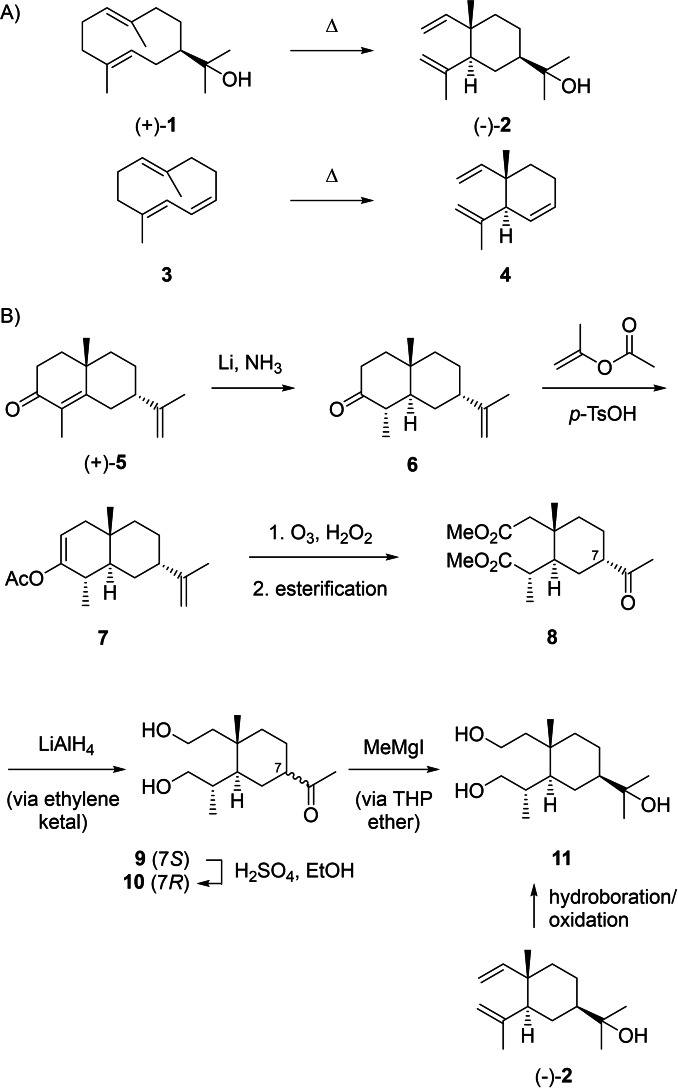
(−)‐Elemol (**2**), the Cope rearrangement product of (+)‐hedycaryol (**1**). A) Cope rearrangements of **1** and pregeijerene (**3**). B) Absolute configuration of (−)‐**2** by chemical correlation to (+)‐10‐*epi*‐α‐cyperone (**5**).

Without detailed knowledge about its structure, in 1916 Semmler and Liao discovered the first monocyclic sesquiterpene alcohol elemol (**2**, Scheme 2A) that was isolated from a fraction of the essential oil of the Philippine tree *Canarium luzonicum* (elemi) obtained by fractional distillation.^[19]^ After establishment of its constitution by Sorm and coworkers,^[20]^ the compound was also found to be the main constituent (60 %) of the essential oil from *Hedycarya angustifolia*, a small tree native to Australia.^[21]^ The missing optical activity of the chiral compound geijerene (**4**), the main constituent in the steam distillates from *Geijera parviflora*, was explained by Jones and Sutherland through their discovery that pregeijerene (**3**) is the true plant natural product that undergoes a Cope rearrangement during compound isolation.^[22]^ Subsequently, the same workers also described **2** as the product of a thermal Cope rearrangement of hedycaryol (**1**).^[23]^ The absolute configuration of **2** has been established independently by chemical correlations to tetrahydrosaussurea lactone^[24]^ and (+)‐10‐*epi*‐α‐cyperone (**5**) in a procedure involving epimerisation of the side chain attached to C7 (Scheme 2B).^[25]^ Reduction of **5** with Li in ammonia gave *trans*‐fused **6** that was converted with isopropenyl acetate and *p*‐TsOH into enol ester **7**, followed by ozonolysis and esterification to **8**. Reduction with LiAlH_4_ via ketalisation with ethylene glycol gave **9** that was easily epimerised under acidic conditions to **10**. Its reaction with MeMgI via protection of the alcohol functions as tetrahydropyranyl (THP) ethers yielded **11**, the same triol that was also obtained through hydroboration and oxidation of **2**.^[25]^

Elemol (**2**) was later reisolated from various plants including *Juniperus sabina* and *J. scopulorum*,[^26^, ^27^] *Chamaecyparis obtusa*,^[28]^ *Citrus sinensis* and *C. nobilis*,[^29^, ^30^, ^31^] *Saussurea lappa*,^[32]^ *Cinnamomum camphora*,^[33]^ *Fokiena hodginsii*,^[34]^ *Calycanthus floridus*,^[35]^ *Bunium cylindricum*,^[36]^ *Gingko biloba*,^[37]^ *Amyris balsamifera*,^[38]^ *Canarium zeylanicum*,^[39]^ *Bothriocloa intermedia*,^[40]^ *Commiphora abyssimica*,^[41]^ *Santolina oblongifolia*,^[42]^ *Cymbopogon proximus*,^[43]^ *Eremophila flaccida*,^[44]^ *Piper ribesioides*,^[45]^ *Monocyclanthus vignei*,^[46]^ *Neocallitropsis pancheri*,^[47]^ *Cryptomeria japonica*,^[48]^ and *Eucalyptus maculata*,^[49]^ which demonstrates the widespread occurrence of **1** in nature. After its first report from *H. angustifolia*,^[23]^ compound **1** was subsequently also isolated from the undistilled oils of the plants *Phebalium ozothamnoides*,^[50]^ *Rubus rosifolius*,^[51]^ *Thujopsis dolabrata*,^[52]^ *Thymus praecox*,^[53]^ *Cryptomeria japonica* and *C. fortunei*,^[54]^ and *Chamaecyparis obtusa*.^[55]^ For the optical rotation of **2** low negative values between [α]_D_=−2 and −9.7 are given in the literature,[^24^, ^26^, ^27^, ^30^, ^32^, ^43^, ^46^, ^48^] while for **1** positive values between [α]_D_=+24.5 and +32.7 were reported.[^23^, ^50^, ^51^, ^52^] The enantiomer (−)‐**1** is only known from the bacterial hedycaryol synthase (HcS) from *Kitasatospora setae* ([α]_D_^25^=−21.3) whose Cope rearrangement gives (+)‐**2** ([α]_D_^25^=+10.0).^[56]^ This finding reflects the observation that also in other cases bacteria and fungi produce the enantiomers of plant terpenes.[^57^, ^58^, ^59^]

**Scheme 3:**
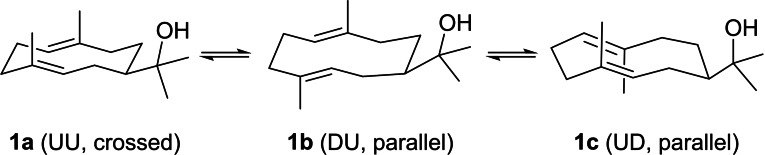
Conformers of **1**. U=Me group at 10‐membered ring up, D=Me group down. „Crossed“ and „parallel“ refers to relative orientations of double bonds.

Because of its strained 10‐membered ring **1** exists as a mixture of three conformers **1 a** with both Me groups attached to the ring up (UU) and crossed double bonds, and **1 b** and **1 c** with parallel double bonds and each one Me group up and one down (DU, UD) (Scheme 3).[^60^, ^61^] Their fairly slow interconversion causes line broadening in the NMR spectra, and therefore the NMR data assignment was a long standing problem that was only recently solved through a ^13^C‐ and stereoselective ^2^H‐labelling approach.^[56]^ Complete NMR data for **2** have also been published.^[47]^ The structure and absolute configuration of (+)‐**1** have been further secured by an enantioselective synthesis from (−)‐guaiol.^[62]^

### 2.2 Biosynthesis, enzymatic and non‐enzymatic cyclisation

**Scheme 4:**
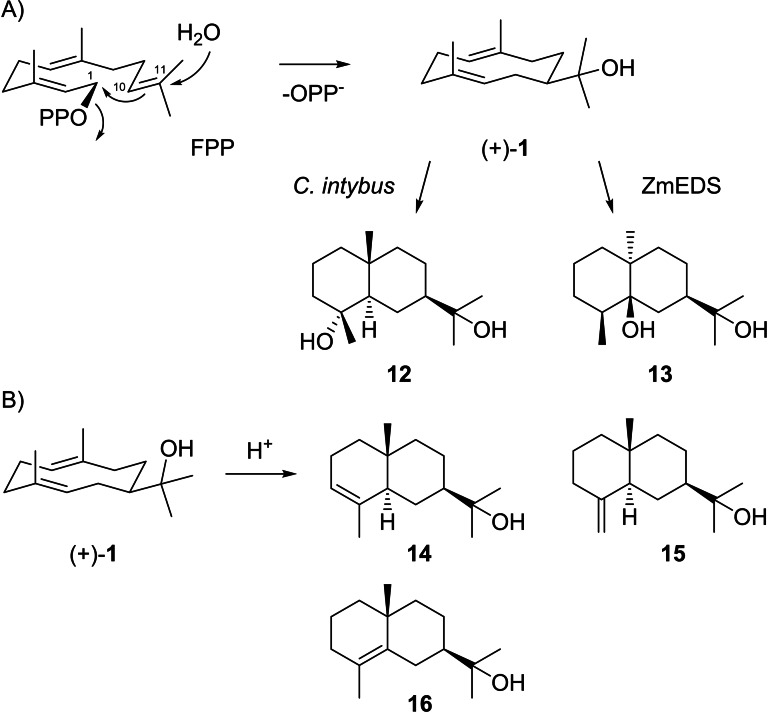
A) Biosynthesis of **1** from FPP and its conversion into **12** and **13**. B) Acid‐catalysed reaction to eudesmols **14**–**16**.

The biosynthesis of **1** by type I terpene synthases proceeds through the abstraction of diphosphate from FPP to initiate a 1,10‐cyclisation and attack of water to C11 (Scheme 4A). Selective hedycaryol synthases for **1** are known from the plants *Populus trichocarpa* (PtTPS7),^[63]^ *Camellia brevistyla* (CbTPS1),^[64]^ and *Liquidambar formosana* (LfTPS01),^[65]^ in all cases with undetermined absolute configuration, and for (−)‐**1** from *Kitasatospora setae*,^[56]^ whose product was initially erroneously assigned as (2*Z*,6*E*)‐hedycaryol; for this bacterial enzyme also a crystal structure is available.^[66]^ In addition, the diterpene synthase VenA from *Streptomyces venezuelae* that converts GGPP into venezuelaene A has a reported side activity with FPP as hedycaryol synthase.^[67]^ For the diterpene synthase spiroviolene synthase from *Streptomyces violens* ^[68]^ ancestral sequence reconstruction resulted in a functional switch to a hedycaryol synthase.^[69]^ As will be discussed in detail in this review article, **1** is an important biosynthetic intermediate, as exemplified by its reported biotransformation into cryptomeridiol (**12**) by a mortared root suspension of chicory (*Cichorium intybus*).^[70]^ Hedycaryol (**1**) is also a proposed intermediate in the biosynthesis of eudesmane‐2α,11‐diol (**13**), the product of the sesquiterpene synthase ZmEDS from *Zea mays*.^[71]^ Herein, the downstream enzymatic cyclisations of **1** are initiated by reprotonation, however, care has to be taken to distinguish enzymatic from non‐enzymatic transformations, as it is well known that **1** can also undergo an efficient non‐enzymatic acid catalysed transannular reaction to yield a mixture mainly composed of α‐, β‐ and γ‐eudesmol (**14** ‐ **16**, Scheme 4B).[^23^, ^72^, ^73^]

**Scheme 5:**
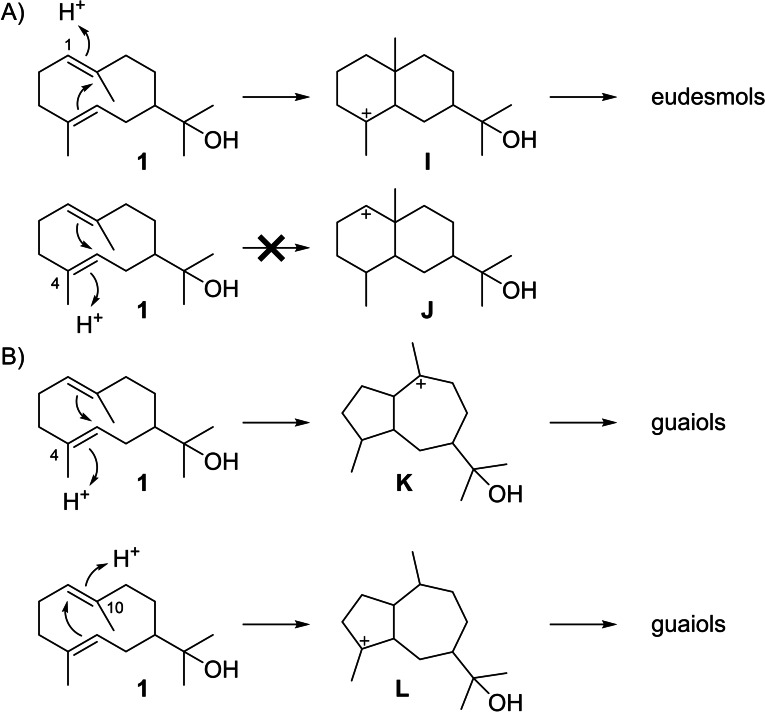
Possible terpene cyclisation modes for **1**.

Terpene synthases can further convert **1** into eudesmols or guaiols through the protonation induced reactions shown in Scheme 5. Reprotonation of **1** at C1 can lead to **I**, the precursor to eudesmols, while the alternative reprotonation at C4 results in the secondary cation **J** that is disfavoured. For guaiols either a protonation at C4 to **K** or at C10 to **L** are possible. The subsequent sections will give a detailed discussion of known compounds arising from **1** via these reactions.

## 3 Eudesmols

### 3.1 Cyclisation modes from hedycaryol to eudesmols

**Scheme 6:**
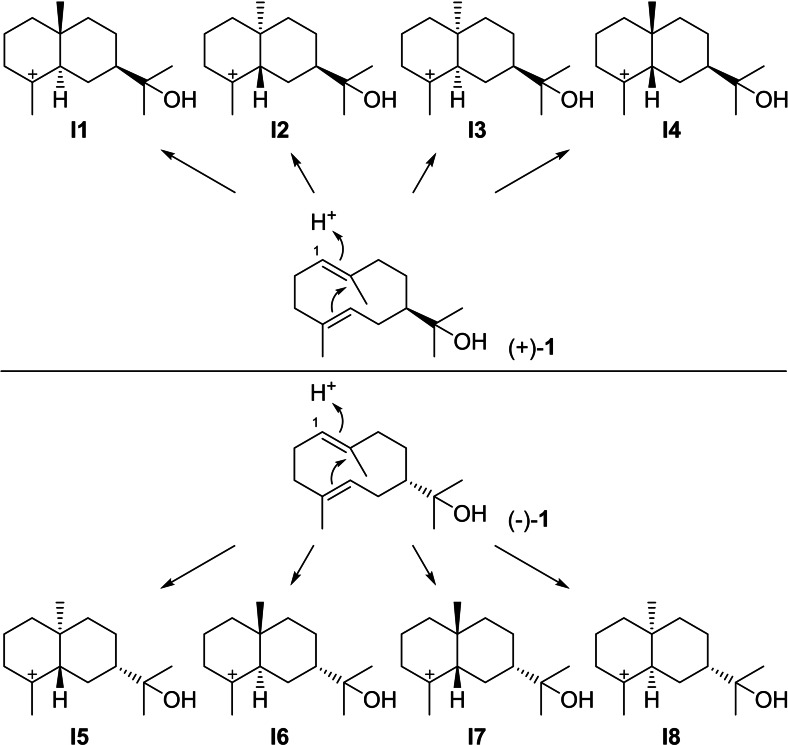
Cyclisation reactions of **1** induced by reprotonation at C1 towards intermediates **I1**‐**I8**.

Eudesmols can arise from (+)‐**1** through protonation at C1 that can induce the cyclisation to the four stereochemically distinct intermediates **I1**‐**I4** (Scheme 6). The corresponding protonation induced cyclisations from (−)‐**1** gives rise to their enantiomers **I5**‐**I8**. All these intermediates can potentially react by three alternative deprotonations, addition of water or intramolecular attack of the hydroxy function at the cation. Further compounds can be formed, if first a 1,2‐hydride shifts occurs that may be followed by skeletal rearrangements.

### 3.2 Eudesmols from cation I1

**Scheme 7:**
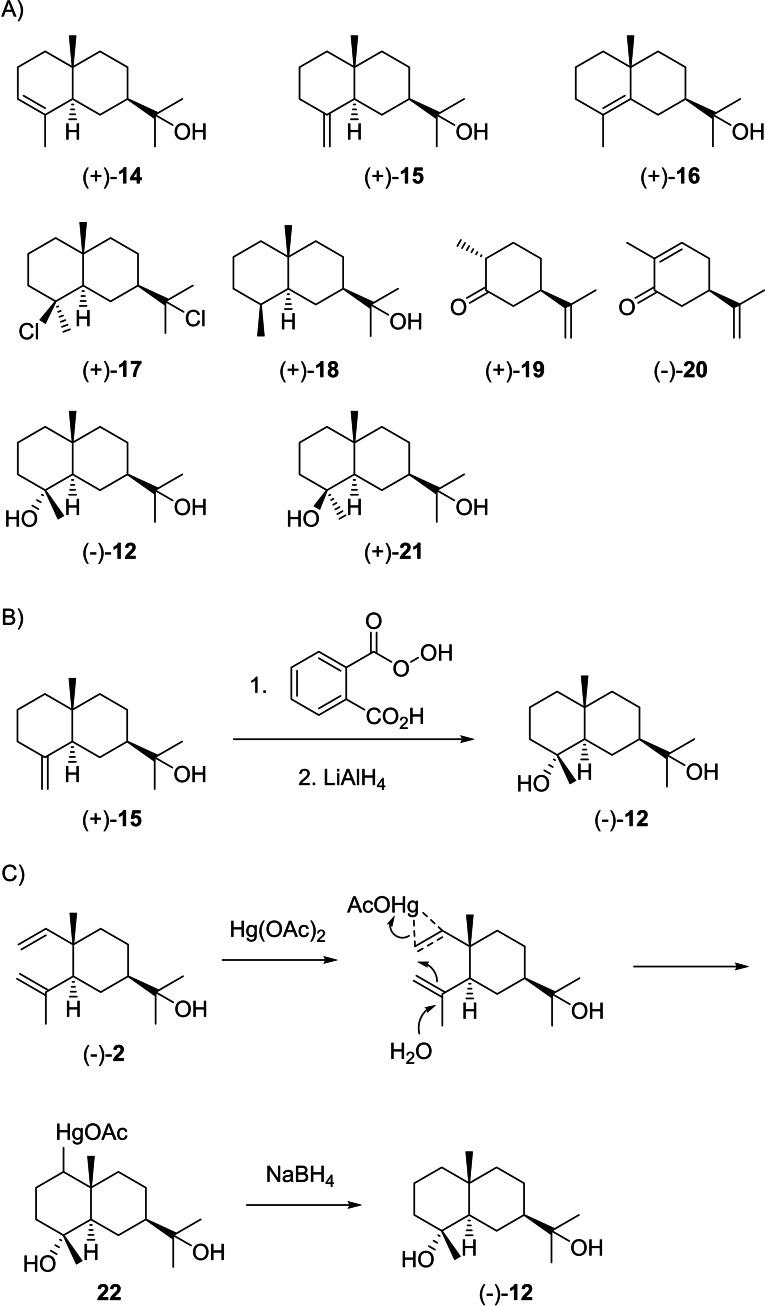
A) Eudesmols derived from **I1** and related compounds. B) Chemical correlation of (+)‐**15** with (−)‐**12** and C) of (−)‐**2** with (−)‐**12**.

Cation **I1** can undergo deprotonations to yield α‐eudesmol (**14**), β‐eudesmol (**15**) or γ‐eudesmol (**16**, Scheme 7A). Ruzicka and coworkers demonstrated that the initially obtained “eudesmol” was a mixture of **14** and **15** of varying composition, which explained the observed variations in melting points and optical rotations.^[74]^ Their separation from *Eucalyptus macarthuri* was first reported by McQuillin and Parrack in 1956. While the separation of **14** and **15** through chromatography on alumina or repeated recrystallisation could not fully be achieved, crystallisation of the 3,5‐dinitrobenzoate esters and their saponification gave access to the pure compounds, establishing positive optical rotations for **14** ([α]_D_=+28.6) and **15** ([α]_D_=+63.8).^[75]^ The same study also reported on the γ‐isomer **16** ([α]_D_=+62.5) that was obtained from (+)‐selinene dihydrochloride (**17**) by elimination and hydrolysis.^[75]^ The absolute configuration of **15** was established by Woodward and coworkers through correlation with the steroids.^[76]^ All three eudesmols **14**–**16** yield the same hydrogenation product (+)‐**18**, confirming their consistent absolute configurations.^[75]^ Further proof for this assignment was obtained by synthesis of eudesmols **14**–**16** from (+)‐dihydrocarvone (**19**).[^77^, ^78^]

The alcohols **14**–**16** were frequently obtained as a mixture from various plants including different *Eucalyptus* species,[^79^, ^80^] *Thuja occidentalis* ^[81]^ and *Phebalium ozothamnoides*,^[50]^ while the pure compounds were isolated from *Callitropsis araucarioides*,^[82]^ *Cordia trichotoma*,^[83]^ and *Cryptomeria japonica*.^[48]^ Finally, **14** was also isolated from the liverwort *Porella perrottetiana*, but in this case the material showed a negative optical rotation ([α]_D_=−6.9).^[84]^ The suggested revision of the optical rotation of **14** with the structure as shown in Scheme 7A from a positive to a negative value, based on a synthetic transformation of (+)‐**15** into (−)‐**14** ^[84]^ conflicts all previous consistent chemical correlations. Also a later study reported a negative optical rotation for **14** obtained by total synthesis from (−)‐carvone (**20**).^[85]^ Despite the unclear situation, the structure of **14** is currently assigned with a negative optical rotation to CAS number 473–16‐5. Final conclusions require further investigations (cf. also discussion in Section 3.6. about *ent*‐**14** derived from **I5**). *Pterocarpus santalinus* is a reported source of pure (+)‐**15**, but its comparably low optical rotation ([α]_D_^30^=+36.0) may point to a contamination with (+)‐**14**.^[86]^ All three compounds **14**–**16** have been isolated from *Neocallitropsis pancheri* with full assignment of ^1^H and ^13^C NMR data.^[47]^

Through the attack of water to the cationic centre in **I1** two diastereoisomeric diols, cryptomeridiol (**12**) and 4‐*epi*‐cryptomeridiol (**21**), can be formed. Cryptomeridiol (**12**) was first isolated from *Widdringtonia dracomontana*, but first only reported as a “diol” of negative optical rotation ([α]_D_=−24).^[87]^ It was subsequently reisolated from *Fokienia hodginsii*, shown to be identical to **12** from *W. dracomontana* by IR spectroscopy and an unchanged melting point upon admixture of an authentic sample, and its structure identified albeit with unspecified configuration at C4. The structural identification mainly relied on the conversion into (+)‐**17** with gaseous HCl and correlated the compound to the same enantiomeric series as the eudesmols.^[88]^ After a third isolation from *Cryptomeria japonica* **12** was named cryptomeridiol and its structure fully assigned by correlation with β‐eudesmol (**15**) that was converted into **12** by epoxidation with monoperphthalic acid and treatment with LiAlH_4_ (Scheme 7B).^[89]^ A more modern version of this synthesis using *m*CPBA for the epoxidation step was published in 1994.^[90]^ Its identity with **12** from *W. dracomontana* and from *F. hodginsii* was not immediately recognised, possibly because of a typographical error in the given name for **12** as “selina‐4,7‐diol”^[88]^ that should read “selina‐4,11‐diol”, but subsequently shown by IR and mixed melting point.^[91]^ Also proximadiol, the anti‐spasmodic principle from *Cymbopogon proximus*,[^92^, ^93^] was later shown to be identical to (−)‐**12**.[^94^, ^95^] Another interesting transformation that secures the absolute configuration of cryptomeridiol is the conversion of (−)‐**2** into (−)‐**12** by oxymercuration and reductive workup (Scheme 7C).^[96]^

The diol **12** is fairly widespread in the plant kingdom and has additionally been isolated from *Artemisia pygmaea*,^[97]^ *Magnolia obovata*,^[98]^ *Drymis winteri*,^[99]^ *Hedychium spicatum*,^[100]^ *Thujopsis dolabrata*,^[101]^ *Carissa edulis*,^[102]^ *Chenopodium graveolens*,^[103]^ *Chamaecyparis pisifera*,^[104]^ *Juglans mandshurica* ^[105]^ and *Achillea clypeolata*,^[106]^ in all cases with a reported negative sign for the optical rotation. Compound (−)‐**12** was also obtained in a biotransformation of synthetic (+)‐**1** with a mortared root suspension of chicory.^[70]^ A terpene synthase for **12** (of undetermined absolute configuration) is known from *Tripterygium wilfordii* (TwCS).^[107]^ However, the surprisingly widespread occurrence of this compound in many plants may also point to a non‐enzymatic formation from (+)‐**1** in an acid catalysed reaction e. g. during chromatographic purifications, especially if water is present,^[70]^ or during steam distillation. This was impressively shown by steam distillation of plant leaves containing (+)‐**1** in the presence of H_2_^18^O, leading to incorporation of the ^18^O‐label into **12** and its epimer **21**.^[108]^ Fully assigned ^1^H‐ and ^13^C NMR data were reported for **12** from the plant *Blumea balsamifera*. For unclear reasons this paper shows the enantiomer of (−)‐**12**.^[109]^

The epimer 4‐*epi*‐cryptomeridiol (**21**) was first isolated from *Amanoa oblongifolia* ([α]_D_=+3.8,^[110]^ in comparison to [α]_D_^25^=+26.1 for the synthetic compound obtained from (+)‐**15**).^[90]^ The same enantiomer (+)‐**21** was later reisolated from *Chamaecyparis pisifera*,^[104]^ *Canarium ovatum*,^[111]^ *Cryptomeria japonica* ^[48]^ and *Citrus hystrix*.^[112]^ Fully assigned ^13^C NMR data have been reported for synthetic **21**.^[90]^

**Scheme 8:**
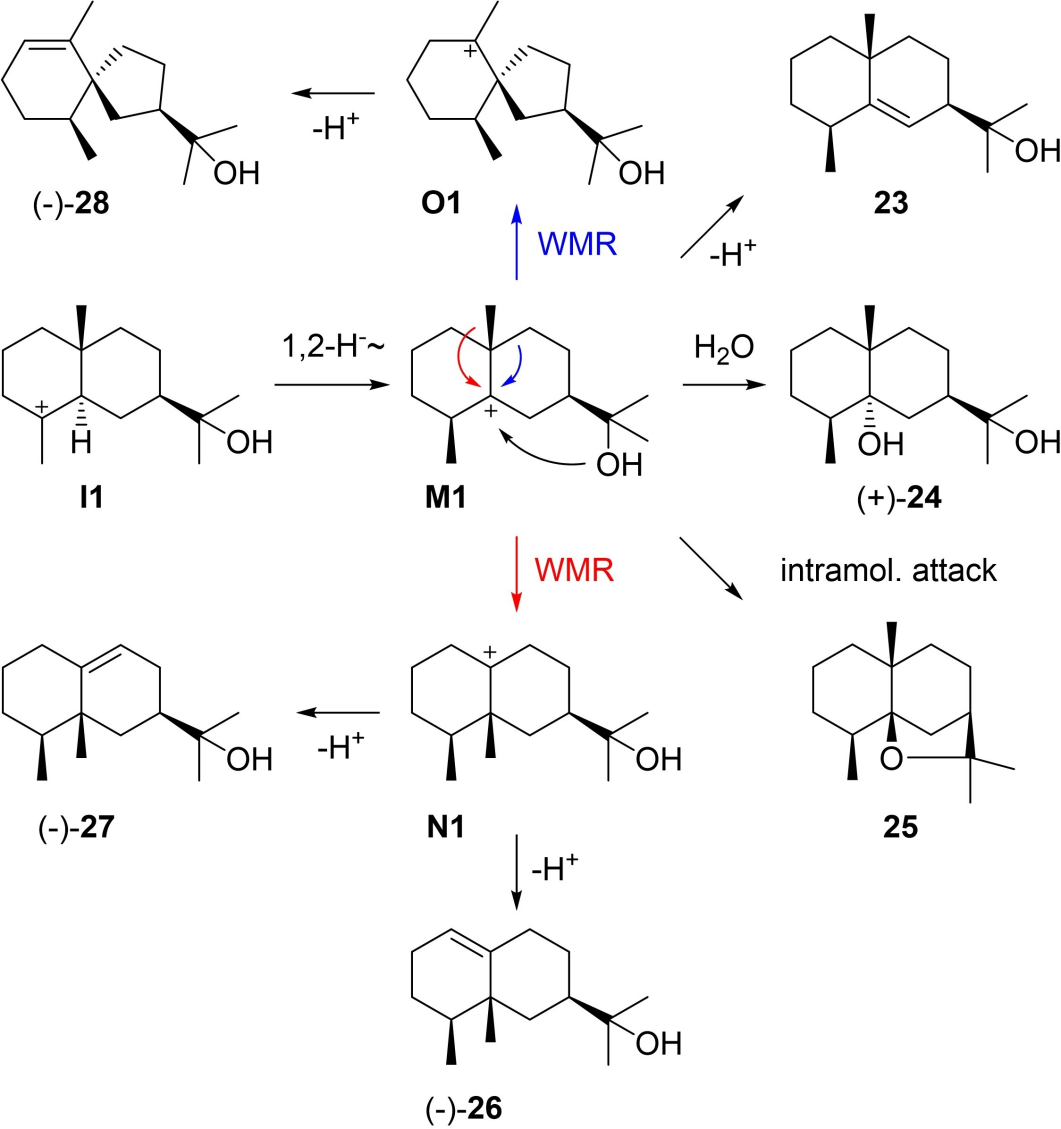
Eudesmols derived from **I1** and 1,2‐hydride shift to **M1**.

**Scheme 9:**
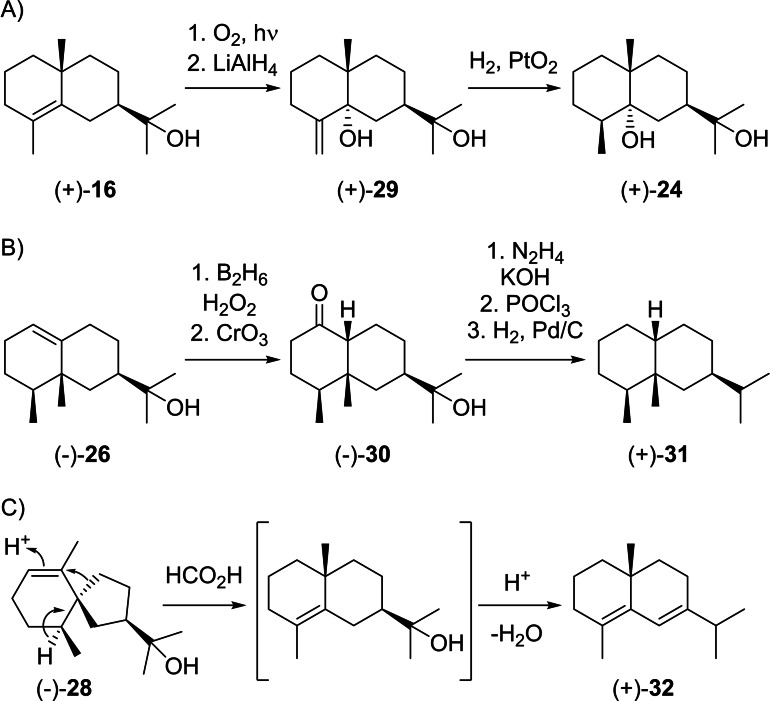
Chemical correlations. A) Synthesis of (+)‐**24** from (+)‐**16**. B) Synthesis of (+)‐**31** from (−)‐**26**. C) Formic acid‐catalysed rearrangement and dehydration of (−)‐**28** to (+)‐**32**.

Cation **I1** can undergo a 1,2‐hydride shift to **M1** that can either react by deprotonation to eudesm‐5‐en‐11‐ol (**23**), by capture with water to (+)‐eudesmane‐5α,11‐diol (**24**), by intramolecular attack of the alcohol function to 4‐*epi*‐*cis*‐dihydroagarofuran (**25**), by Wagner‐Meerwein rearrangement (WMR) to **N1** and deprotonation to (−)‐eremoligenol (**26**) or its isomer **27**, or by WMR to **O1** and deprotonation to (−)‐hinesol (**28**, Scheme 8). Only few reports are available for **23** that was first isolated from *Helichrysum italicum* ^[113]^ and later from *Bulnesia sarmientoi*.^[114]^ Unfortunately, both studies did not report on the optical rotation of **23** and its absolute configuration has not formally been established, while fully assigned NMR data were given in both cases.[^113^, ^114^] The diol **24** was first obtained synthetically from (+)‐γ‐eudesmol (**16**) by photochemical oxidation and reduction of the allyl hydroperoxide, followed by catalytic hydrogenation (Scheme 9A), establishing its positive optical rotation ([α]_D_=+41.9).^[115]^ The same enantiomer was later reported with completely assigned NMR data from *Cryptomeria japonica*.^[48]^ The epimer of **24** with 5β‐hydroxy group has only been obtained by synthesis,^[62]^ but not from natural sources. The ether **25** was reported from *Cedrelopsis grevei* ^[116]^ and from *Pseuduvaria froggattii*, from which it was named froggatt ether.^[117]^ Both studies gave fully assigned NMR data, but neither reported the optical rotation nor established the absolute configuration.[^116^, ^117^]

The rearranged compound eremoligenol (**26**) was first isolated from *Ligularia fischeri* ([α]_D_=−93.5) and its absolute configuration was established by correlation to (+)‐eremophilane (**31**) through a sequence of hydroboration and oxidation to the ketole **30**, followed by Huang‐Minlon reduction, dehydration and catalytic hydrogenation (Scheme 9B).^[118]^ The compound was later reisolated from *Euryops sulcatus* ^[119]^ and *Oreodaphne porosa*.^[120]^ The isomer **27** was first obtained as a synthetic material^[121]^ followed by its isolation from *Alpinia japonica* ([α]_D_=−14.9).^[122]^ (−)‐Hinesol (**28**) was first reported from *Atractylodes lancea* ([α]_D_=−40.2) and shown to be a constituent of „atractylol“ that was initially believed to be a pure compound.^[123]^ Its structure was initially wrongly assigned,^[124]^ but later corrected with a suggested absolute configuration based on its co‐occurrence with (+)‐β‐eudesmol (**15**).^[125]^ This assignment was later confirmed by a correlation with (+)‐δ‐selinene (**32**) that was obtained from **28** by formic acid catalysed rearrangement and dehydration (Scheme 9C), albeit not in pure form,^[126]^ and by an enantioselective synthesis of (−)‐**28**.^[127]^ Hinesol shows an antitrypanosomal activity against *Trypanosoma brucei*.^[128]^

### 3.3 Eudesmols from cation I2

**Scheme 10:**
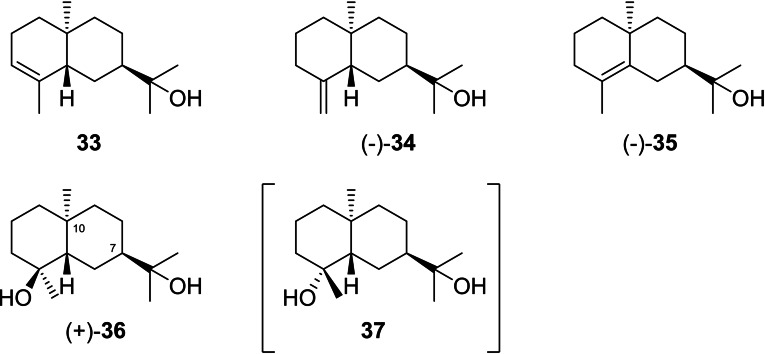
Eudesmols derived from **I2**. Compound **37** in brackets is unknown.

Cation **I2** could potentially lead to the alcohols **33**–**35** by deprotonation or to the diols **36** and **37** by addition of water (Scheme 10). For **33** only a synthesis of the racemate has been reported,^[129]^ while **34** ([α]_D_^25^=−17.5) has been synthesised enantioselectively from (+)‐intermedeol,^[130]^ but both compounds are not known from natural sources. Also 10‐*epi*‐γ‐eudesmol (**35**) was first obtained by synthesis from dihydrocarvone (+)‐**19**, unfortunately without reporting the optical rotation of **35**,^[131]^ but the first isolation paper mentions the identity of (−)‐**35** from vetiver oil (*Vetiveria zizanioides*) and the synthetic material.^[132]^ The compound was also isolated from *Amyris balsamifera*,^[38]^ *Aquilaria malaccensis* ([α]_D_=−68.8),^[133]^ *Alpinia japonica*,^[122]^ *Hedychium spicatum* ^[134]^ and *Bursera graveolens*.^[135]^

The diol **36** was also first synthesised,^[136]^ followed by an isolation from *Ursinia trifida*,^[137]^ in both cases without mentioning the optical rotation. At the same time the isolation of a compound from *Pluchea arguta* with same ^13^C NMR data (apart from C4, this is likely a typographical error), but with a *cis*‐decalin structure (10‐*epi*‐**36**) was reported ([α]_D_^29^=+66.66).^[138]^ This erroneous structural assignment was later corrected based on a total synthesis of (+)‐**36** ([α]_D_^29^=+73.3) from (+)‐dihydrocarvone (**19**).^[139]^ Pterodondiol from *Laggera pterodonta* for which initially a structure with 7*S* configuration was published,[^140^, ^141^] is identical to **36** (with its 7*R* configuration), as was later demonstrated by X‐ray crystallography.^[142]^ Compound **36** is additionally known from *Goniothalamus tapisoides*.^[143]^ ^13^C NMR data of **36** have been published in CDCl_3_ ^[137]^ and in C_5_D_5_N.^[140]^ Compound **37** is unknown.

**Scheme 11:**
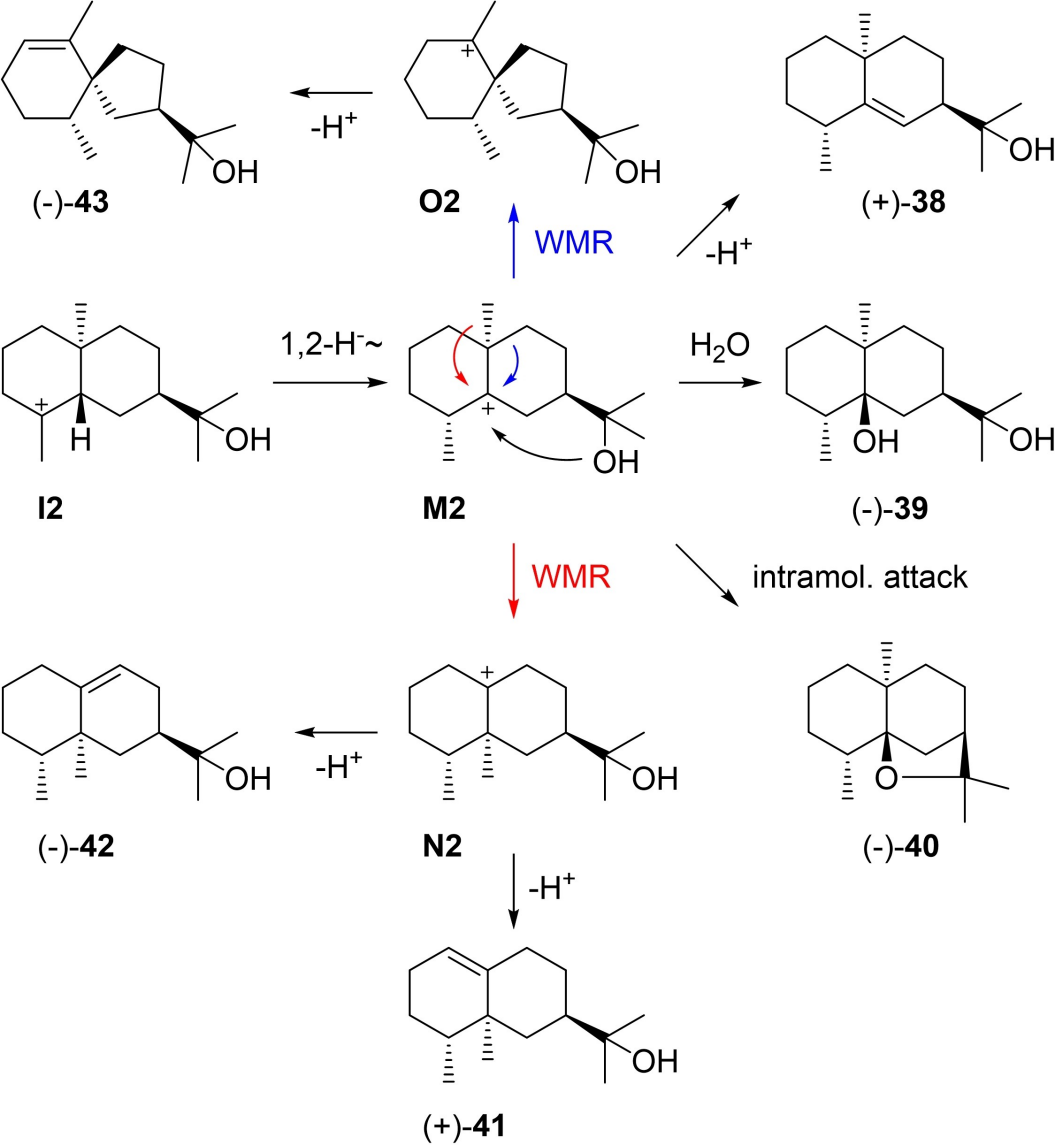
Eudesmols derived from **I2** and 1,2‐hydride shift to **M2**.

Rearranged compounds from **I2** (Scheme 11) can be accessed by a 1,2‐hydride shift to **M2**, from which a deprotonation leads to (+)‐rosifoliol (**38**), a capture with water to (−)‐**39**, and the intramolecular attack of the hydroxy function to (−)‐dihydro‐β‐agarofuran (**40**). A methyl migration to **N2** and deprotonation can result in (+)‐valerianol (**41**) or (−)‐jinkoheremol (**42**), while ring contraction to **O2** and deprotonation lead to (−)‐agarospirol (**43**). Most of these compounds are fairly widespread.

**Scheme 12:**
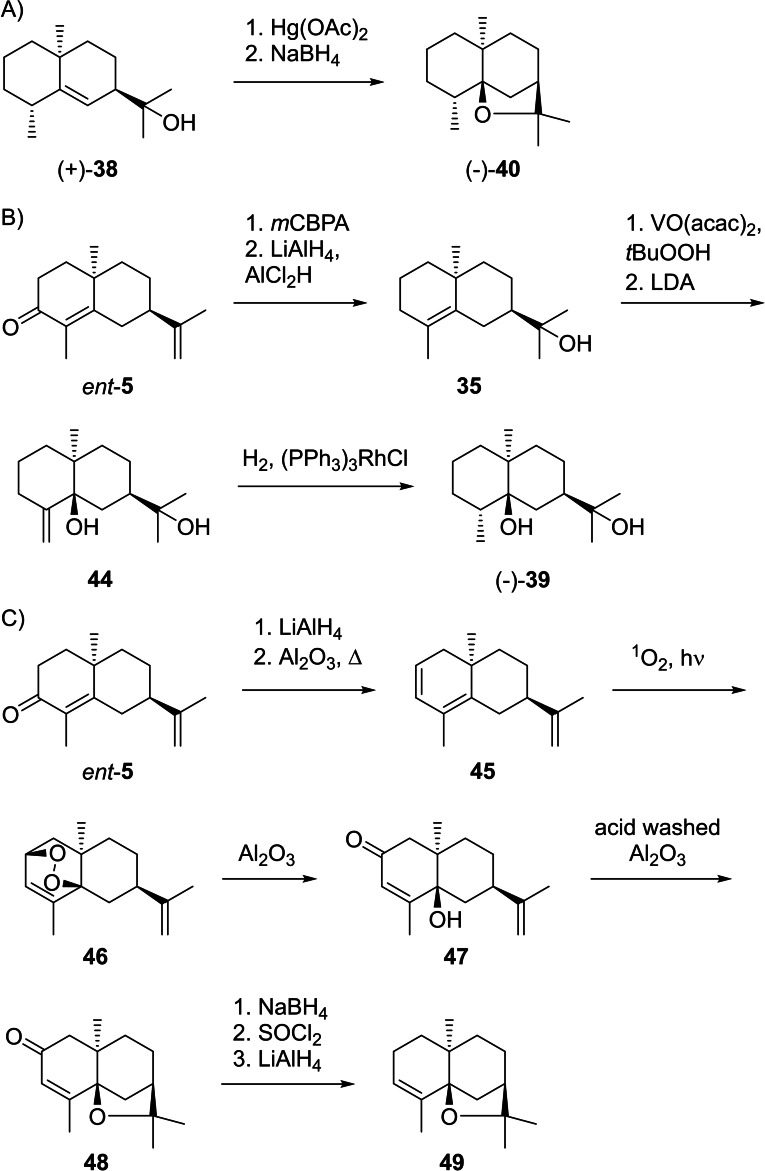
Chemical correlations. A) Synthesis of (−)‐**40** from (−)‐**38**. B) Synthesis of (−)‐**39** from (−)‐*ent*‐**5**. C) Synthesis of α‐agarofuran (**49**) from (−)‐10‐*epi*‐α‐cyperone (*ent*‐**5**).

Rosifoliol (**38**), [α]_D_=+105, was first isolated from *Rubus rosifolius*,^[144]^ after its possible formation along the lines of Scheme 11 had been proposed.^[145]^ Its structure and absolute configuration were established by correlation with (−)‐**40** (Scheme 12A),^[51]^ and also the X‐ray crystal structure has been obtained.^[146]^ The alcohol **38** was also found in *Phonus arborescens*, but this time with a reported negative optical rotation that was not commented on ([α]_D_^20^=−17.1).^[147]^ Also the ^13^C NMR data differ substantially,[^144^, ^147^] leaving doubt if the material from *P. arborescens* is indeed identical to the originally isolated rosifoliol. The diol **39** was so far only isolated from *Alpinia japonica* ([α]_D_=−21.8)^[148]^ and its structure was secured by synthesis from (−)‐10‐*epi*‐α‐cyperone (*ent*‐**5**) that proceeded by epoxidation with *m*CPBA and epoxide opening with ketone reduction using LiAlH_4_ and AlCl_2_H to yield 10‐*epi*‐γ‐eudesmol (**35**, Scheme 12B). Selective β‐epoxidation with VO(acac)_2_ and *t*BuOOH followed by epoxide opening with LDA gave **44** that was catalytically hydrogenated with Wilkonson's catalyst to obtain (−)‐**39** ([α]_D_^10^=−46.2).^[149]^

Dihydro‐β‐agarofuran (**40**, [α]_D_^30^=−77.01) was first isolated from fungus‐infected agarwood (*Aquillaria agallocha*) with unknown configuration at C4 and the configurations at C5 and C7 determined wrongly.^[150]^ The structure was later revised based on a synthesis from *ent*‐**5** that gave the diene **45** upon reduction with LiAlH_4_ and pyrolysis in the presence of basic alumina (Scheme 12C). Photosensitised oxygenation to peroxide **46** was followed by isomerisation to the hydroxy ketone **47** under mildly basic conditions. Treatment with acid‐washed Al_2_O_3_ resulted in ring closure to **48**, that upon reduction to a stereoisomeric mixture of allyl alcohols with NaBH_4_, conversion into the allyl chlorides with SOCl_2_ and reduction with LiAlH_4_ gave α‐agarofuran (**49**).^[151]^

**Scheme 13:**
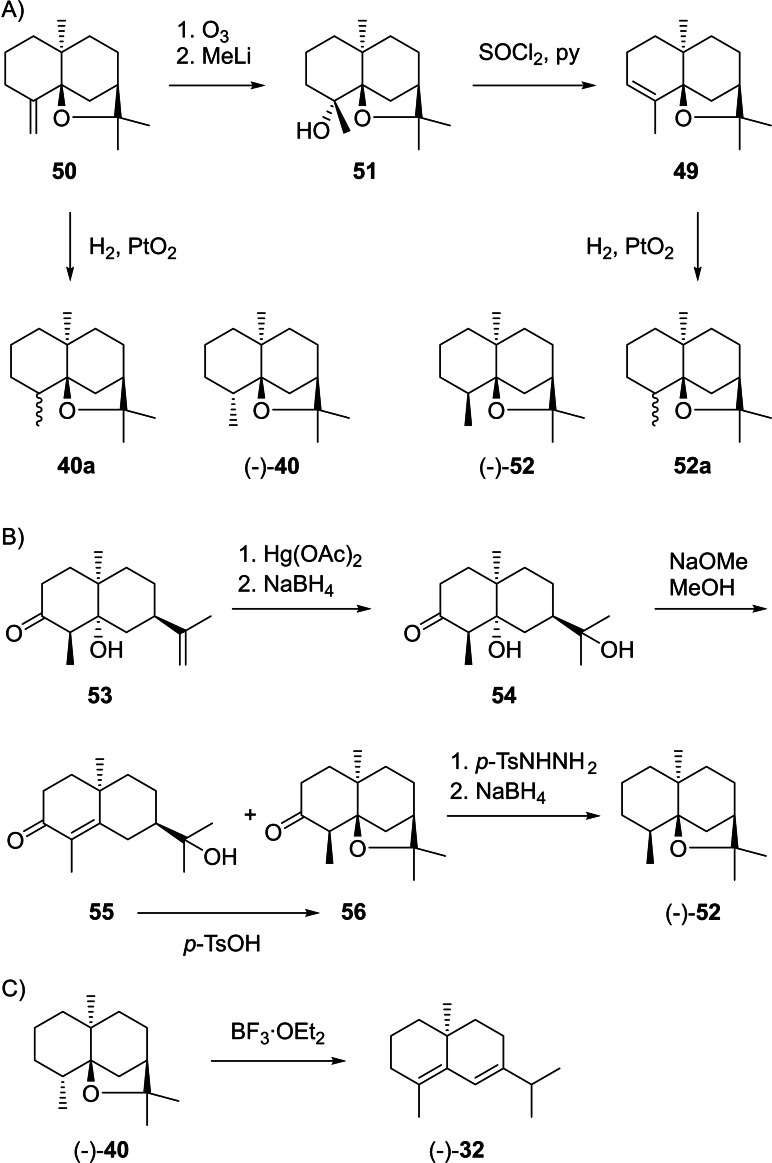
Chemical correlations. A) Conversion of **50** into **49** and catalytic hydrogenations. B) Synthesis of (−)‐**52** from **53**. C) Absolute configuration of (−)‐**40** by correlation with (−)‐δ‐selinene (**32**).

**Scheme 14:**
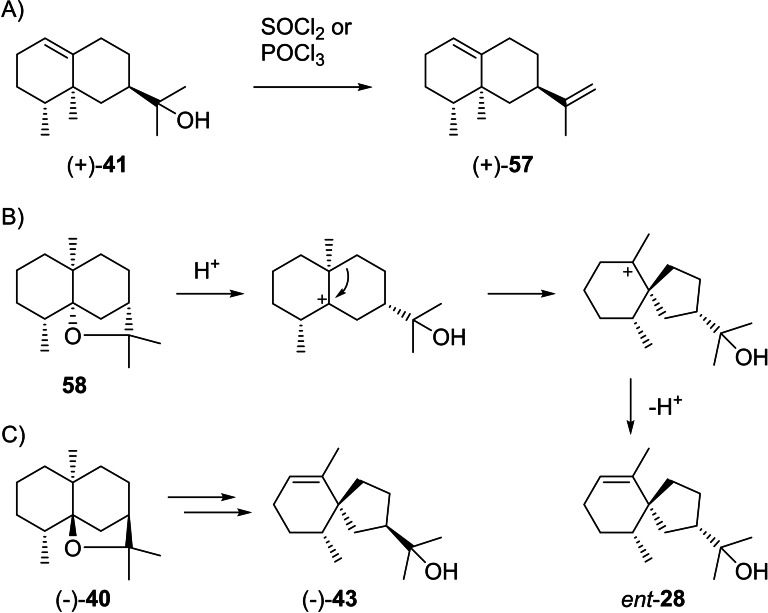
Chemical correlations. A) Dehydration of (+)‐**41** to (+)‐**57**. B) Hypothetical structure for agarospirol (*ent*‐**28**) based on an assumed biosynthetic relation to dihydro‐β‐agarofuran with the initially reported structure of **58**. C) Revised structure of **40** for dihydro‐β‐agarofuran and analogous biosynthetic relation to the correct structure **43** of agarospirol.

At this stage the previous work had shown that **49** can be obtained from β‐agarofuran (**50**) by ozonolysis and addition of MeLi to **51**, followed by dehydration with SOCl_2_ in pyridine (Scheme 13A).[^150^, ^152^] It was also known that the catalytic hydrogenation of **49** and **50** leads to materials with slightly different properties, with the compound obtained from **50** being identical to natural (−)‐**40**. The two compounds **40 a** and **52 a** were suggested to be stereoisomers, but their configurations at C4 were unclear.^[150]^ A later erroneous correlation with valencene through biotransformation resulted in a confusion of these stereoisomers,[^153^, ^154^] but the situation was ultimately resolved by a synthesis of (−)‐isodihydroagarofuran (**52**) from **53** (Scheme 13B).^[155]^ This route proceeded through oxymercuration to **54**. Treatment with NaOMe in MeOH gave a mixture of mainly **55** and small amounts of **56**, with **55** being convertible into **56** under acid catalysis with *p*‐TsOH. Reduction with *p*‐toluenesulfonyl hydrazine and NaBH_4_ resulted in (−)‐**52** that was identical to the product obtained by catalytic hydrogenation of **49**, and consequently also the structure of **40** (=4‐*epi*‐**52**) was secured. The absolute configuration of (−)‐**40** was evident from its correlation to (−)‐δ‐selinene formed upon treatment with BF_3_ etherate (Scheme 13C).^[150]^ The ether (−)‐**40** was also isolated from *Galbanum* resin,^[156]^ *Alpinia japonica*,^[122]^ *Laggera alata* ^[157]^ and *Vetiveria zizanioides*.^[158]^

(+)‐Valerianol (**41**) was first isolated from *Valeriana officinalis* ([α]_D_^20^=+134) and its absolute configuration was established by dehydration with SOCl_2_ or POCl_3_, yielding a hydrocarbon that was identical with (+)‐valencene (**57**, Scheme 14A).^[159]^ It is also known from *Amyris balsamifera* ^[38]^ and agarwood,^[160]^ and is the main product of the G411 A enzyme variant of *Zea mays* eudesmanediol synthase (ZmEDS).^[71]^ Kusunol that was reported from *Cinnamomum camphora* is identical to (+)‐**41**.^[161]^ (−)‐Jinkoheremol (**42**) was first isolated from agarwood and its structure was determined by NMR spectroscopy. Further proof for the assigned structure was given by catalytic hydrogenation that yielded a mixture of the same epimeric dihydro‐compounds as obtained from **41**. The absolute configuration was tentatively assigned by comparison of its optical rotation ([α]_D_=−66) to values for structurally similar compounds,^[160]^ but has not been formally established by chemical correlation. (−)‐Agarospirol (**43**) was first isolated from *Aquilaria agollocha* ([α]_D_^27^=−5.7) with a suggested structure of *ent*‐hinesol (*ent*‐**28**), based on a biosynthetic relation to dihydro‐β‐agarofuran with the at that time assumed structure of **58** (Scheme 14B). The same paper suggested **43** as an alternative stereochemical representation.^[162]^ Notably, after the structural revision of dihydro‐β‐agarofuran to **40**[^151^, ^155^] an analogous biosynthetic relation can indeed explain **43** (Scheme 14C). A synthesis of (*rac*)‐**28** also excluded this structure for agarospirol,^[163]^ while later syntheses of (*rac*)‐ and (−)‐**43** confirmed its structure and absolute configuration.[^164^, ^165^] A later report about agarwood constituents claims a reisolation of (−)‐**43**, but shows the structure of *ent*‐**28**.^[160]^ Neuroleptic properties have been described for **42** and **43** in mice which may be responsible for the sedative effects of agarwood.^[166]^

### 3.4 Eudesmols from cation I3

**Scheme 15:**
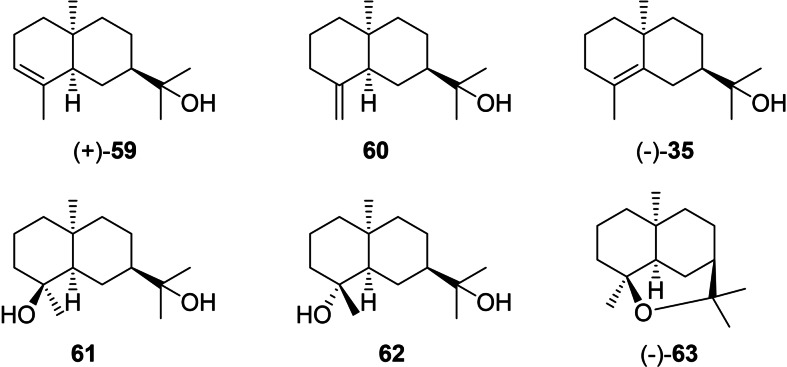
Eudesmols derived from **I3**.

**Scheme 16:**
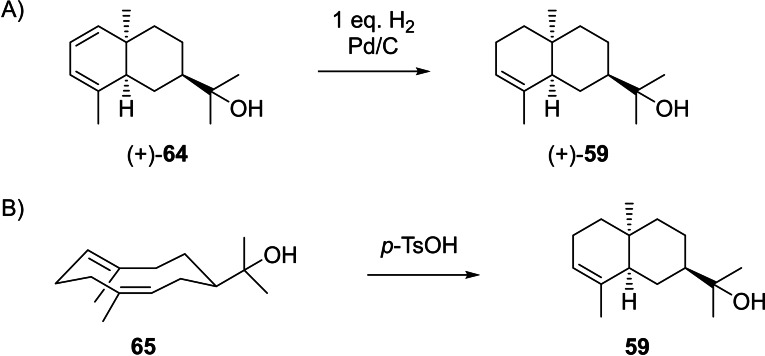
Chemical correlations. A) Catalytic hydrogenation of (+)‐**64**. B) Acid‐catalysed conversion of (*Z*,*E*)‐hedycaryol (**65**).

The structures of the eudesmols that can directly be formed from **I3** by deprotonation (**59**, **60** and **35**), capture with water (**61** and **62**) or intramolecular attack of the alcohol to the cation (**63**) are shown in Scheme 15. Compound **35** has already been discussed above as a deprotonation product from **I2** (Scheme 10).

(+)‐Dihydrooccidentalol (**59**), [α]_D_^24^=+59.2, is not known as a natural product, but was obtained by catalytic hydrogenation from (+)‐occidentalol (**64**, Scheme 16A), a constituent of *Thuja occidentalis* ^[167]^ for which the structure was assigned by detailed analysis of coupling constants in the ^1^H NMR spectrum.^[168]^ The compound is also formed from (*Z*,*E*)‐hedycaryol (**65**) upon acid catalysed transannular reaction (Scheme 16B).^[169]^ 10‐*epi*‐β‐Eudesmol (**60**) has been isolated from *Bulnesia sarmientoi* with fully established structure by 2‐dimensional NMR techniques,^[114]^ but neither the optical rotation has been reported nor the absolute configuration has been assigned. The diols **61** and **62** are unknown from natural sources and have only been obtained by synthesis of their racemates.^[170]^ The ether (−)‐4,11‐epoxy‐*cis*‐eudesmane (**63**, [α]_D_^28^=−22)^[171]^ is a major constituent of the frontal gland secretions of the termite *Amitermes evuncifer*.^[172]^ Its structure was first correctly assigned based on a series of microreactions^[172]^ and later confirmed by an enantioselective synthesis from (−)‐carvone (**20**).^[171]^ Compound **63** was later also isolated from *Amitermes excellens* ^[173]^ and from *A. minimus*, in which case the paper erroneously shows the opposite absolute configuration, but still reports a negative optical rotation ([α]_D_^26^=−34).^[174]^ Interestingly, (−)‐**63** has a repellent activity against the ant *Crematogaster californica*.^[174]^ The same ether **63** is also known from the plant *Phonus arborescens*.^[147]^

**Scheme 17:**
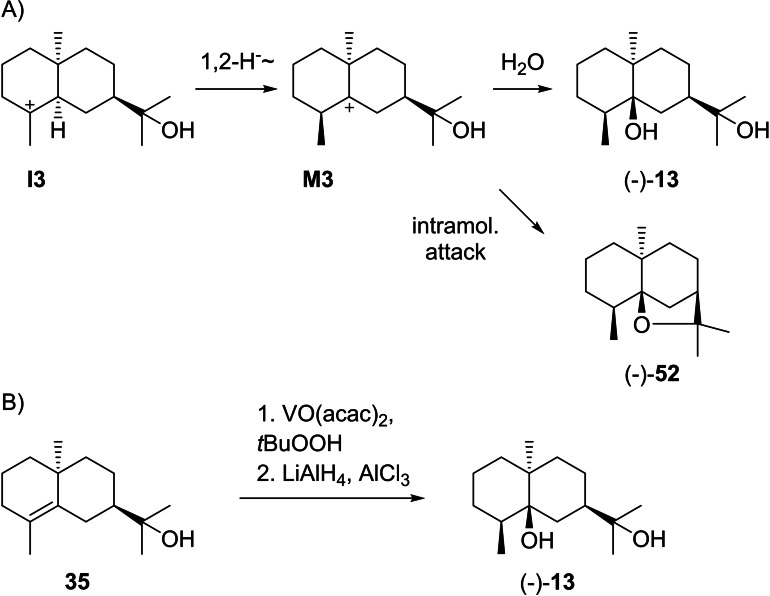
A) Eudesmols derived from **I3** and 1,2‐hydride shift to **M3**. B) Synthesis of (−)‐**13**.

Further compounds from **I3** (Scheme 17A) can be reached by a 1,2‐hydride shift to **M3** and capture with water to **13** or intramolecular attack of the alcohol to (−)‐**52** for which structure elucidation has already been discussed above. The diol **13** ([α]_D_^25^=−9.0) was so far only isolated from *Cymbopogon distans* with structure elucidation based on NMR spectroscopy and X‐ray crystallography,^[175]^ and is the main product of *Zea mays* eudesmanediol synthase (ZmEDS).^[176]^ The absolute configuration was evident through a synthesis from **35** (prepared as shown in Scheme 12) by epoxidation and reductive epoxide opening (Scheme 17B).^[149]^ Isodihydroagarofuran (**52**), also named α‐dihydroagarofuran, was isolated from *Phonus arborescens*,^[147]^ *Bursera graveolens*,^[135]^ *Bulnesia sarmientoi*,^[177]^ and identified in the cyanobacterium *Calothrix* by GC/MS in comparison to standards of **52** and its stereoisomer **40**, albeit without determination of absolute configuration.^[178]^

### 3.5 Eudesmols from cation I4

**Scheme 18:**
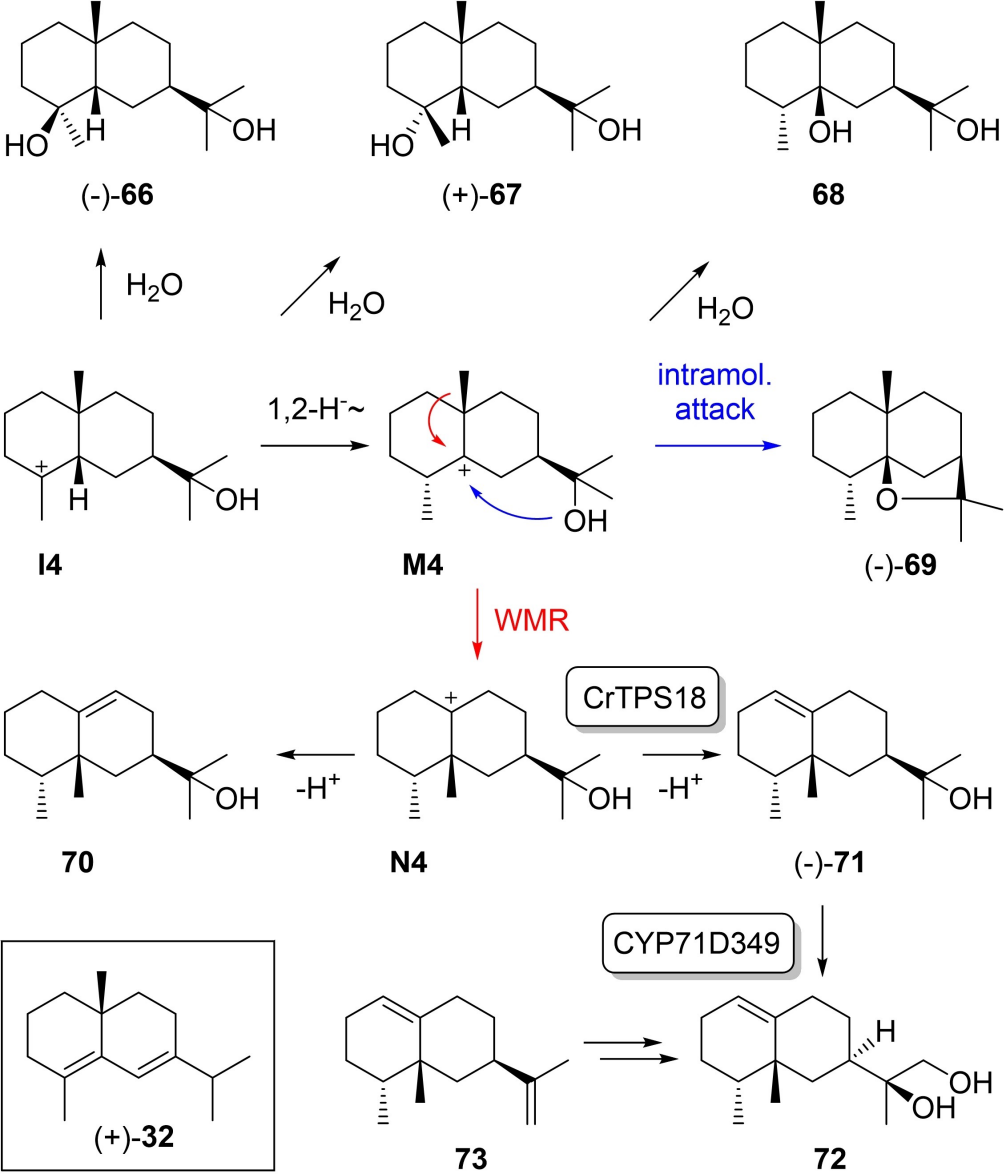
Eudesmols derived from **I4**.

Little is known about eudesmols from cation **I4** (Scheme 18). The alcohols **66** ([α]_D_^20^=−41.1) and **67** ([α]_D_^20^=+21.16) were only obtained by synthesis.^[90]^ The erroneous assignment of structure **66** to a sesquiterpene diol from *Pluchea arguta* and its structural revision to **36** have been discussed above.[^138^, ^139^] Compounds that are accessible after 1,2‐hydride shift to **M4** include the diol **68** that is unknown from natural sources, but has been obtained by synthesis together with its C4 epimer without further structural assignment regarding the stereochemistry at C4.^[115]^ Intramolecular attack of the alcohol function to the cation in **M4** gives access to (−)‐*cis*‐dihydroagarofuran (**69**) that was so far only isolated from *Prostanthera ovalifolia* ([α]_D_^25^=−87.6). Its relative configuration was determined by 2‐dimensional NMR techniques and direct comparison to its stereoisomers **40** and **52**, while the absolute configuration was evident from its dehydration to (+)‐δ‐selinene (**32**, boxed in Scheme 18).^[179]^

Methyl group migration from **M4** to **N4** and deprotonation gives access to (−)‐5‐*epi*‐jinkoheremol (**71**, [α]_D_^25^=−15) for which recently a terpene synthase from *Catharanthus roseus* (CrTPS18) was discovered.^[180]^ The absolute configuration of **71** was determined by a comparison of measured to calculated ECD curves. Notably, **71** was shown to be the biosynthetic precursor of debneyol (**72**) by a genetically clustered cytochrome P450 monooxygenase (CYP71D349),^[180]^ which is in contrast to the earlier findings for the biosynthesis of **72** that showed incorporation of radioactivity from the sesquiterpene hydrocarbon 5‐*epi*‐aristolochene (**73**).^[181]^ Alternatively, **N4** can be deprotonated to **70**, which is unknown as a natural product, but the racemic compound has been synthesised.^[182]^

### 3.6 Eudesmols from cation I5

**Scheme 19:**
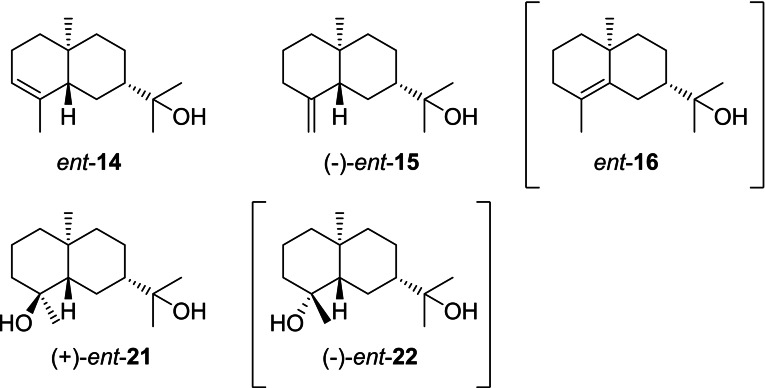
Eudesmols that can directly arise from **I5**. Compounds in brackets are unknown.

Generally, the number of reports on compounds from the enantiomeric series derived from (−)‐hedycaryol through cations **I5** ‐ **I8** is much lower than those discussed above for (+)‐hedycaryol derivatives. Compounds that could biosynthetically directly arise from **I5** (Scheme 19) include *ent*‐α‐eudesmol (*ent*‐**14**) for which only one synthetic report is available. Herein, the absolute configuration was secured by MoKα X‐ray crystallography of the *p*‐bromobenzoate‐epoxide of *ent*‐**14** (Flack parameter: 0.030(3)) and the optical rotation of *ent*‐**14** was found to be positive ([α]_D_^25^=+6.4)^[183]^ which supports the suggested revision of the signs of optical rotation for the enantiomers of **14**.^[84]^ The freshwater fungus *Beltriana rhombica* is a source of *ent*‐**15** ([α]_D_^29^=−37.9),^[184]^ and (+)‐cryptomeridiol (*ent*‐**21**) has been reported from the cypress *Chamaecyparis obtusa*,^[185]^ while *ent*‐**16** and *ent*‐**22** are unknown. No natural products obtained from **I5** through 1,2‐hydride shift and eventually skeletal rearrangement are known.

### 3.7 Eudesmols from cation I6

**Scheme 20:**
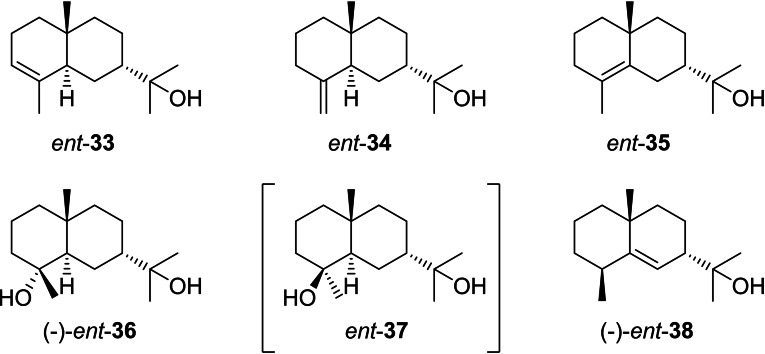
Eudesmols derived from **I6**. Compound *ent*‐**37** in brackets is unknown.

Compounds that can directly arise from **I6** are summarised on Scheme 20. The sesquiterpene alcohol 7‐*epi*‐α‐eudesmol (*ent*‐**33**) was first claimed from *Amyris balsamifera*. The absolute configuration was concluded from the positive optical rotation ([α]_D_=+10),^[186]^ but since at that time no reference data of either enantiomer had been reported, the reason for this assignment is unclear. Notably, all other related compounds from this plant have the usual 7*R* configuration.^[38]^ 7‐*epi*‐γ‐Eudesmol (*ent*‐**35**) was first reported with a negative optical rotation ([α]_D_^25^=−15) from *Cryptomeria japonica*.^[48]^ This work describes structure elucidation by NMR, but does also not explain the reasoning for the assignment of absolute configuration. Subsequently, *ent*‐**33** was also reported from *Laggera alata* without stating the optical rotation, together with *ent*‐**34** and *ent*‐**35** for which again negative optical rotations were given.^[157]^ However, this conflicts previous assignments based on enantioselective syntheses of (−)‐**34** and, from (+)‐dihydrocarvone, of (−)‐**35** (cf. Section 3.3.).[^130^, ^131^, ^132^] The situation becomes even more confusing, because a later synthesis study reported the transformation of (−)‐dihydrocarvone into (−)‐*ent*‐**35** ([α]_D_^10^=−30.1).^[187]^ Taken together, the assignments of optical rotations especially to the enantiomers of **35** are doubtful and await future clarification. 7‐*epi*‐α‐Eudesmol (**33**) has also been observed as the product of a bacterial sesquiterpene synthase from *Streptomyces viridochromogenes*.[^58^, ^188^] Homologs of this enzyme can be found in many streptomycetes.^[189]^ The absolute configuration of **33** from 7‐*epi*‐α‐eudesmol synthase is undetermined, but the enantiomer *ent*‐**33** would possibly fit best for a bacterial compound as bacteria often produce the opposite enantiomer as observed in plants.

For isodonsesquitin A from *Isodon grandifolia* the structure of *ent*‐**36** was assigned, but the positive optical rotation ([α]_D_^26^=+24.6) is in conflict with this assignment,^[190]^ because a total synthesis of both enantiomers gave [α]_D_^29^=−66.7 for *ent*‐**36** and [α]_D_^29^=+73.3 for **36**. The measurements also revealed a strong concentration dependency of these data, but always gave the same sign of optical rotation for the same enantiomer.^[139]^ Unfortunately, the isolation paper from *I. grandifolia* did not further discuss the problem of absolute configuration assignment,^[190]^ and thus the assignment may likely be in error in this study. After a first assignment of the structure of **67** to a diol from *Pluchea arguta* ^[138]^ a revision based on synthetic work suggested the compound to be *ent*‐**36**,^[90]^ but after synthesis of both enantiomers it was ultimately demonstrated that **36** is the correct structure.^[139]^ *Pluchea quitoc* is also a reported source of *ent*‐**36**,^[191]^ giving a references to its isolation and first structural revision.[^90^, ^138^] With the correction of the absolute configuration for the compound from *P. arguta* ^[139]^ it must be concluded that also *P. quitoc* is a producer of **36**. Taken together, despite some discussions about *ent*‐**36** from natural sources in the literature, it seems that this compound is not known as a natural product. Also no reports are available for its C4 epimer *ent*‐**37**. (−)‐*ent*‐Rosifoliol (*ent*‐**38**) can arise from **I6** by 1,2‐hydride shift and deprotonation and has been described from the liverwort *Calypogeia muelleriana*.^[192]^

### 3.8 Eudesmols from cation I7

**Scheme 21:**
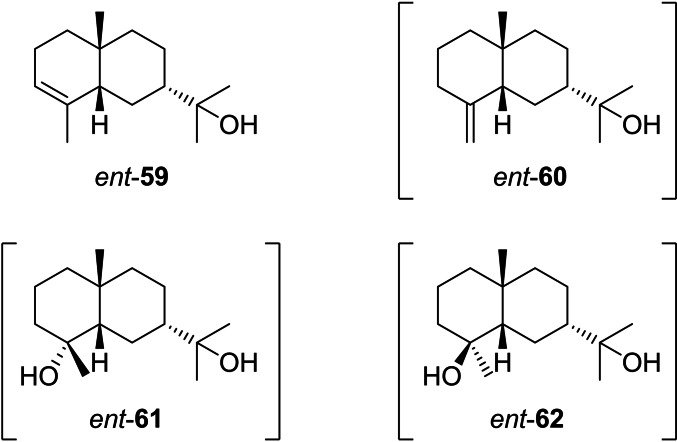
Eudesmols derived from **I7**. Compounds in brackets are unknown.

Eudesmols potentially arising from cation **I7** are shown in Scheme 21. Starting with a report about the composition of the essential oil from *Elionurus elegans*,^[193]^ compound *ent*‐**59** (“5‐*epi*‐7‐*epi*‐α‐eudesmol”) is mentioned in several GC/MS based studies, but has never been isolated, which leaves doubt about the absolute configuration assignment and most if not all these studies may indeed have detected **59** instead. This view is in line with the fact that also neither *ent*‐**60**, *ent*‐**61** and *ent*‐**62** nor any compounds arising from **I7** by 1,2‐hydride shift and eventually skeletal rearrangement have ever been reported. In summary, no secure reports about natural products from **I7** are available.

### 3.9 Eudesmols from cation I8

**Scheme 22:**
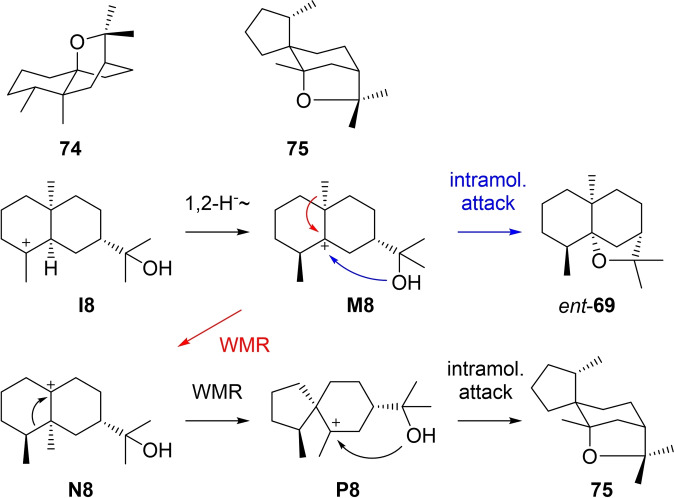
Eudesmols derived from **I8**.

Only very little is known about eudesmol derivatives arising through cation **I8** (Scheme 22). The knowledge is basically limited to the fungal phytotoxin hypodoratoxide. After the initially assigned structure of **74** ^[194]^ was corrected to that of **75**,^[195]^ the biosynthesis was investigated through feeding experiments with isotopically labelled precursors. Starting from **I8**, a 1,2‐hydride shift leads to **M8** that can be deprotonated to *ent*‐**69**, a cometabolite of **75** in *Hypomyces odoratus*. A methyl migration to **N8**, skeletal rearrangement to **P8** and intramolecular attack of the alcohol function to the cation result in **75**.^[195]^ The absolute configurations of **69** and **75** in *H. odoratus* have not firmly been established.

## 4 Guaiols

### 4.1 Cyclisation of hedycaryol by protonation at C4

**Scheme 23:**
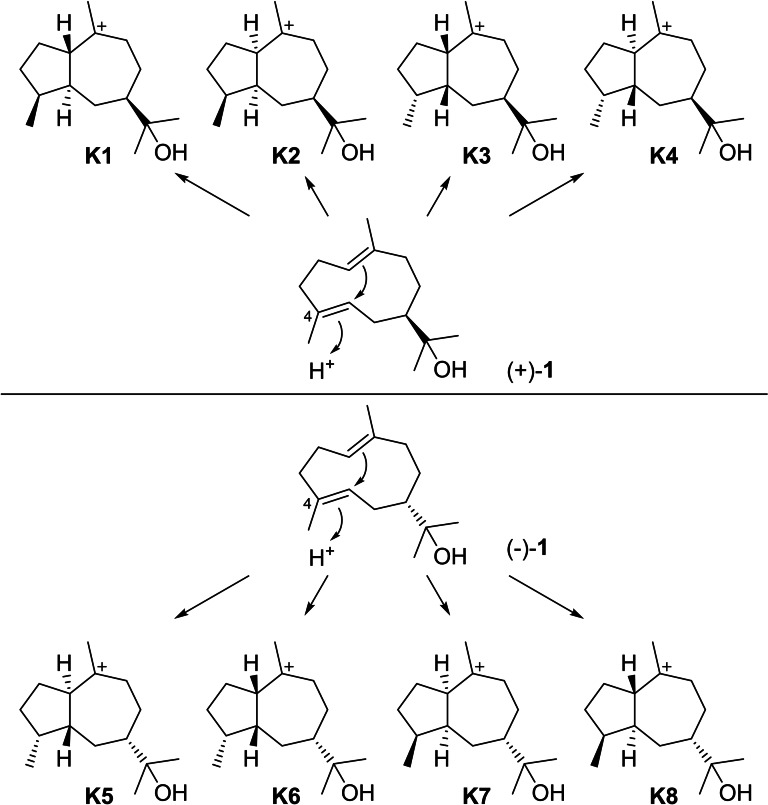
Cyclisation reactions of **1** induced by reprotonation at C4 towards intermediates **K1**–**8**.

Hedycaryol (+)‐**1** can undergo cyclisations through protonation at C4 towards four stereoisomeric intermediates **K1**–**K4** (Scheme 23). The series of opposite enantiomers **K5**–**K8** is analogously accessible through protonation induced cyclisations from (−)‐**1**, but no natural products with unequivocally established absolute configurations from these intermediates with 7*S* configuration appear in the literature. In all cases H5 and Me15 are *trans* to each other because the addition to the *E* configured C4=C5 double bond of hedycaryol is necessarily *anti*. The following sections discuss all known natural products that can be formed from the **K** stereoisomers either directly by deprotonation, capture with water or intramolecular attack of the alcohol function, or after hydride shifts.

### 4.2 Guaiols from cation K1

**Scheme 24:**
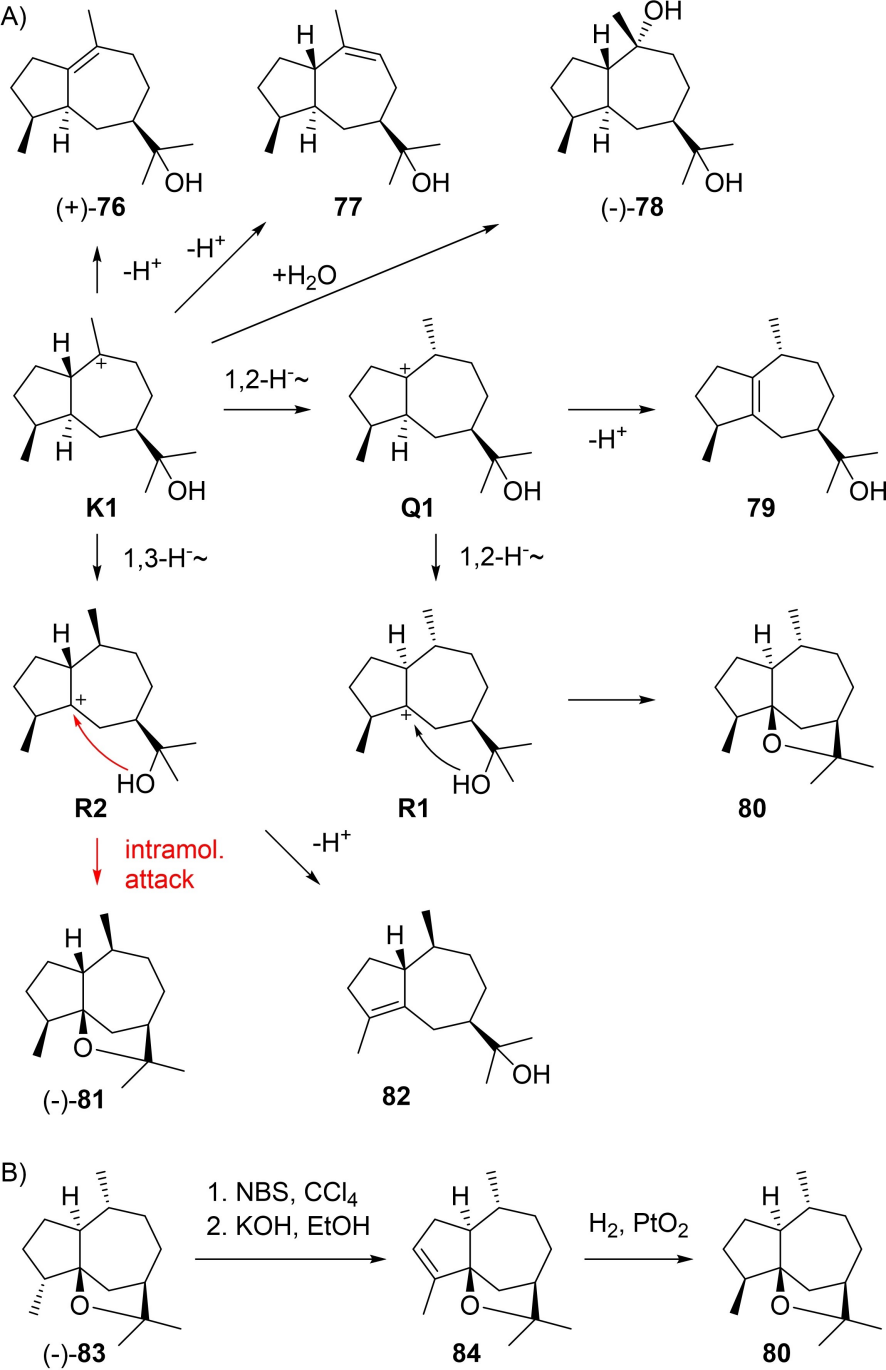
A) Guaiols derived from **K1**. B) Conversion of the natural product **83** into its epimer **80**.

**Scheme 25:**
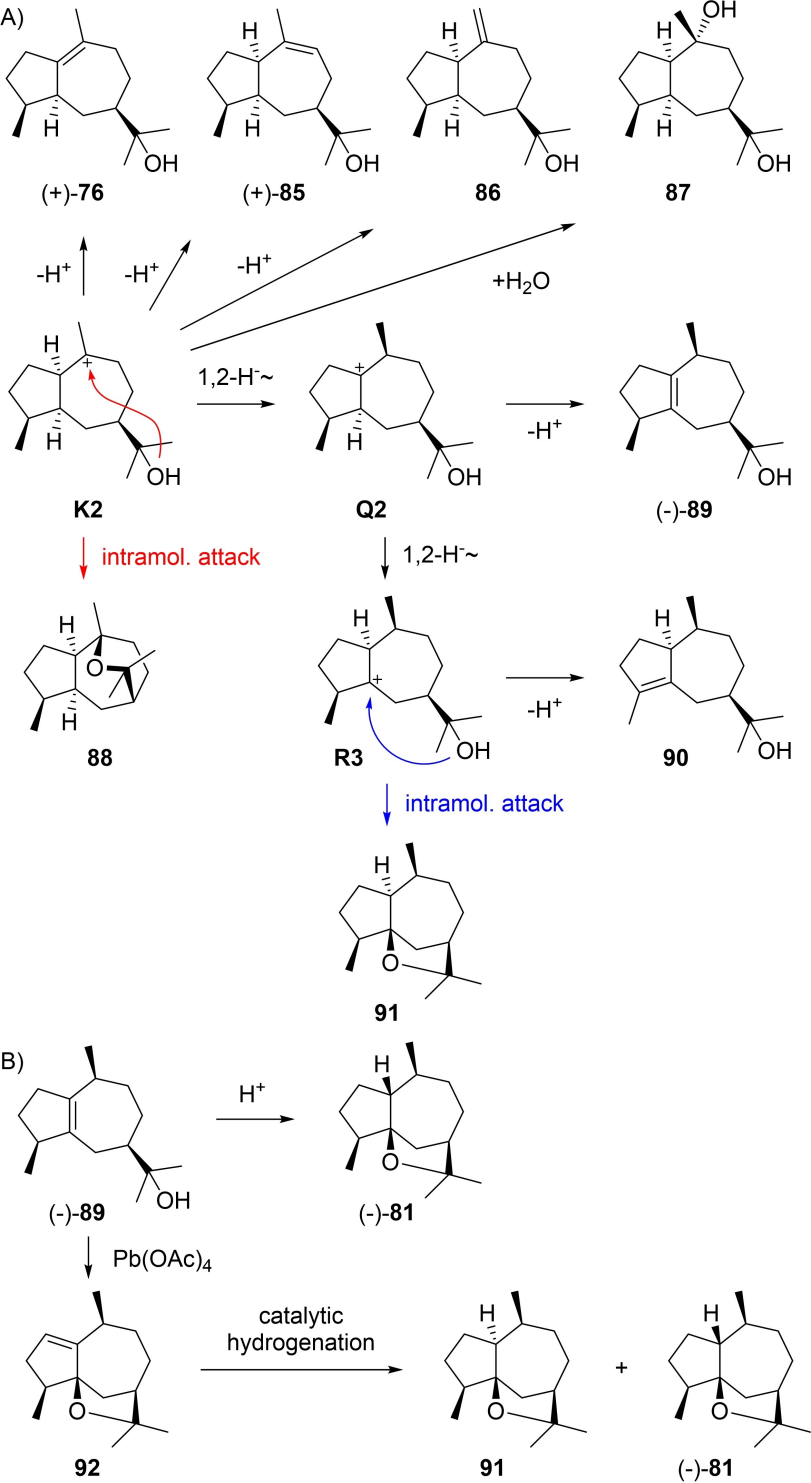
A) Guaiols derived from **K2**. B) Chemical correlations of **89** with **81** and **91**.

Guaiols that can be formed directly from **K1** are shown in Scheme 24A. (+)‐Bulnesol (**76**) from guaiacwood oil ([α]_D_^20^=+3.8)^[196]^ is one of the most important representatives of the class of guaiols. Its structure was elucidated by Sorm in a correlation to guaiol (**89**, Scheme 25A) that yielded the same hydrogenation product as **76**.[^196^, ^197^, ^198^] It was later also isolated from *Galbanum* resin^[199]^ and *Neocallitropsis pancheri*,^[47]^ and a sesquiterpene synthase from *Thapsia laciniata* for the production of **76** and **89** as main products (TlTPS509) with compound isolation by preparative GC and NMR based structure elucidation was described.^[200]^ The alcohol 5αH‐guai‐9‐en‐11‐ol (**77**) was recently reported from guaiacwood oil,^[114]^ while the diol (−)‐**78** ([α]_D_^25^=−25.0) is known from the extremophilic fungus *Pithomyces* isolated from a mine waste pit.^[201]^ The absolute configuration of **78** has not formally been established yet. Starting from **K1** a 1,2‐hydride shift to **Q1** and deprotonation explain **79** that has also recently been found in guaiacwood oil.^[114]^ The ether **80** can arise from **Q1** by a second 1,2‐hydride shift to **R1** and intramolecular attack of the alcohol function, but is only known as a synthetic compound that was obtained from its 4‐epimer (−)‐**83**, a known natural product from *Ligularia* ([α]_578_=−45, Scheme 24B).^[202]^ Bromination at C4 with NBS and elimination gave **84** that upon catalytic hydrogenation yielded **80**,^[202]^ thereby completing the set of all eight stereoisomers with 7*R* configuration (for discussion of other stereoisomers see below). A 1,3‐hydride shift from **K1** to **R2** and deprotonation yield the alcohol **82** from guaiacwood oil,^[114]^ while ring closure gives guaioxide (**81**) that will be discussed in detail in the next section.

### 4.3 Guaiols from cation K2

Compounds from **K2** are summarised in Scheme 25A. As an alternative to its formation from **K1**, bulnesol (**76**) could also be formed from **K2** by deprotonation, which may better explain its co‐occurrence with guaiol (**89**), the lead compound from the class of hedycaryol derived 5–7 membered bicyclic sesquiterpene alcohols, that can also be formed from **K2** by 1,2‐hydride shift to **Q2** and deprotonation. Guaiol was first described from guaiacwood by Gandurin ([α]_D_^25^=−26.64) as a bicyclic tertiary alcohol with one double bond.^[203]^ The compound is widespread and has also been isolated from *Callitris intratropica*,^[204]^ *Eucalyptus maculata*,^[205]^ *Drimys lanceolata*,^[206]^ *Cinnamomum camphora*,^[207]^ *Callitris columellaris*,^[208]^ *Guillonea scabra*,^[209]^ *Thapsia villosa*,^[210]^ *Canarium luzonicum* (Manila elemi),^[211]^ *Murraya gleinei*,^[212]^ *Neocallitropsis pancheri*,^[213]^ *Eriostemon fitzgeraldii*,^[214]^ *Ferula ferulioides* ^[215]^ and *Uvaria puguensis*,^[216]^ and is a product of the above mentioned terpene synthase TlTPS509 from *Thapsia laciniata*.^[200]^ After establishment of its constitution,^[217]^ the absolute configuration was clarified by chemical correlation.[^196^, ^198^, ^218^, ^219^]

Other known compounds that can directly arise from **K2** include *cis*‐guai‐9‐en‐11‐ol (**85**) from *Galbanum* resin ([α]_D_^20^=+4.9)^[156]^ and from guaiacwood oil that is also a source of 1αH,5αH‐guai‐10(14)‐en‐11‐ol (**86**) and 10,11‐epoxyguaiane (**88**).[^114^, ^177^] The diol **87** was first isolated from *Leuceria floribunda* with the relative configuration secured by NOE experiments,^[220]^ and later reported again from *Jatropha curcas*.^[221]^ Starting from **Q2**, a second 1,2‐hydride shift to **R3** and deprotonation leads to **90**. This compound is known from guaiacwood oil^[114]^ and has been synthesised from guaiol (**89**).^[222]^ (−)‐Guaioxide (**81**, [α]_D_^24^=−38.2) is easily formed by acid treatment of **89** (Scheme 25B).[^223^, ^224^] It has also been isolated from guaiacwood oil, but may have been formed during the isolation process.^[177]^ Its hypothetical biosynthesis requires a 1,3‐hydride shift from **K1** to **R2** and intramolecular attack of the alcohol function (Scheme 24A). The stereoisomer 1‐*epi*‐guaioxide (**91**) can arise analogously from **R3**, but is not known as a natural product (Scheme 25A). Both compounds have been synthesised from **89** by oxidation with Pb(OAc)_4_ to yield **92**, followed by catalytic hydrogenation to **91** and (−)‐**81** (Scheme 25B).^[225]^ Guaioxide (**81**) has also been correlated to dihydroguaiol, the hydrogenation product of **89**, by a combination of microbial and chemical transformations.^[226]^

### 4.4 Guaiols from cation K3

**Scheme 26:**
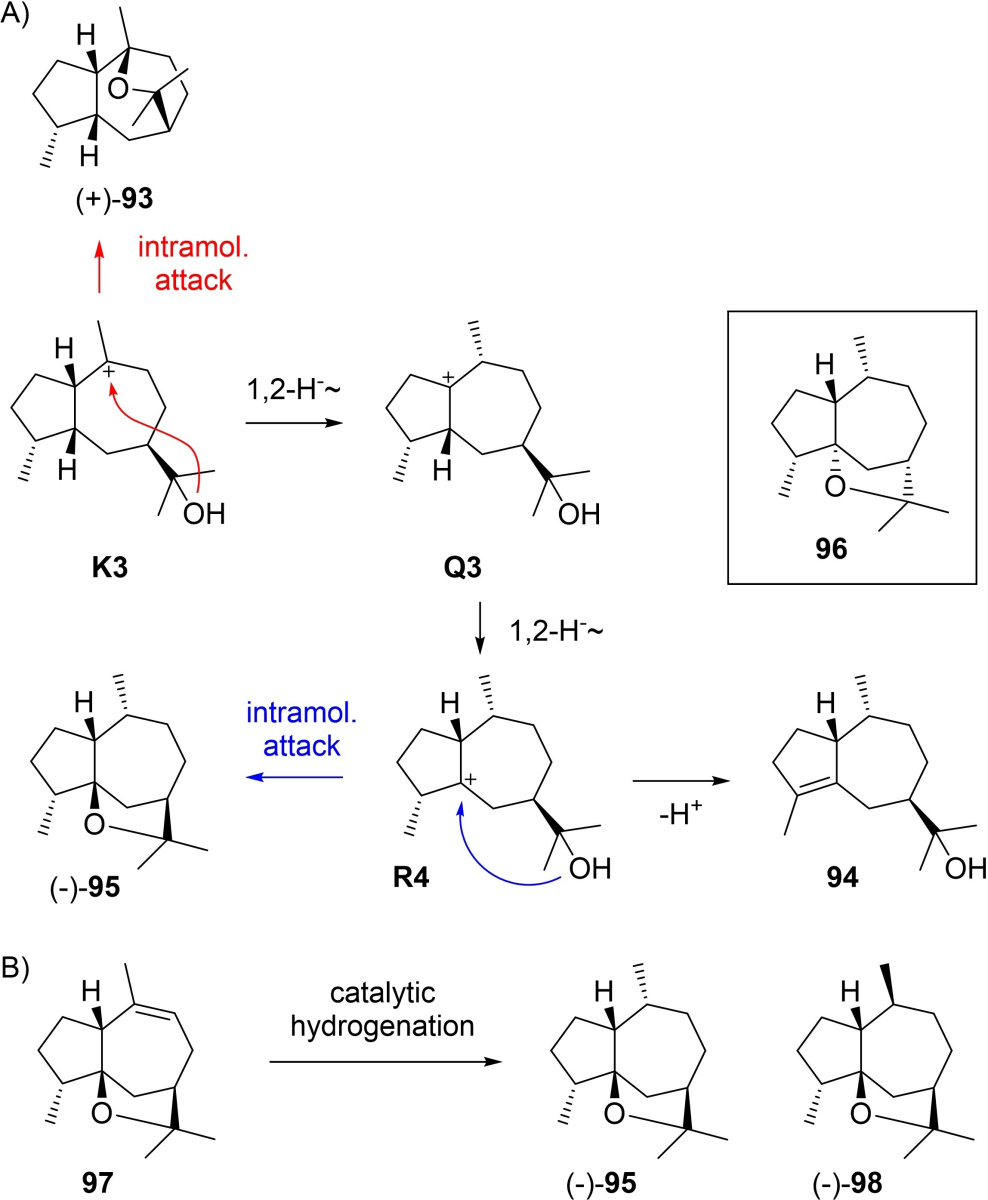
A) Guaiols derived from **K3**. B) Catalytic hydrogenation of **97** yields the epimers **95** and **98**.

Guiaols from **K3** include (+)‐isokessane (**93**) by intramolecular attack of the alcohol (Scheme 26A). This compound has been isolated from *Rubus rosifolius* ([α]_D_=+19.2) and its structure was elucidated by one and two‐dimensional NMR spectroscopy.^[227]^ The alcohol **94** is known from guaiacwood oil^[114]^ and can arise through a sequence of two 1,2‐hydride shifts to **Q3** and **R4**, followed by deprotonation. Alternatively, **R4** can react by ring closure to (−)‐10‐*epi*‐liguloxide (**95**) that has been isolated from *Ligularia* ([α]_D_=−3.5).^[228]^ For this compound initially the structure of **96** (box in Scheme 26A) was assigned, but a later structural revision of liguloxide (**98**) showed the requirement of a structural revision also of **95**,^[229]^ because the two compounds are epimers as they are simultaneously formed by catalytic hydrogenation of **97** (Scheme 26B).^[228]^

### 4.5 Guaiols from cation K4

**Scheme 27:**
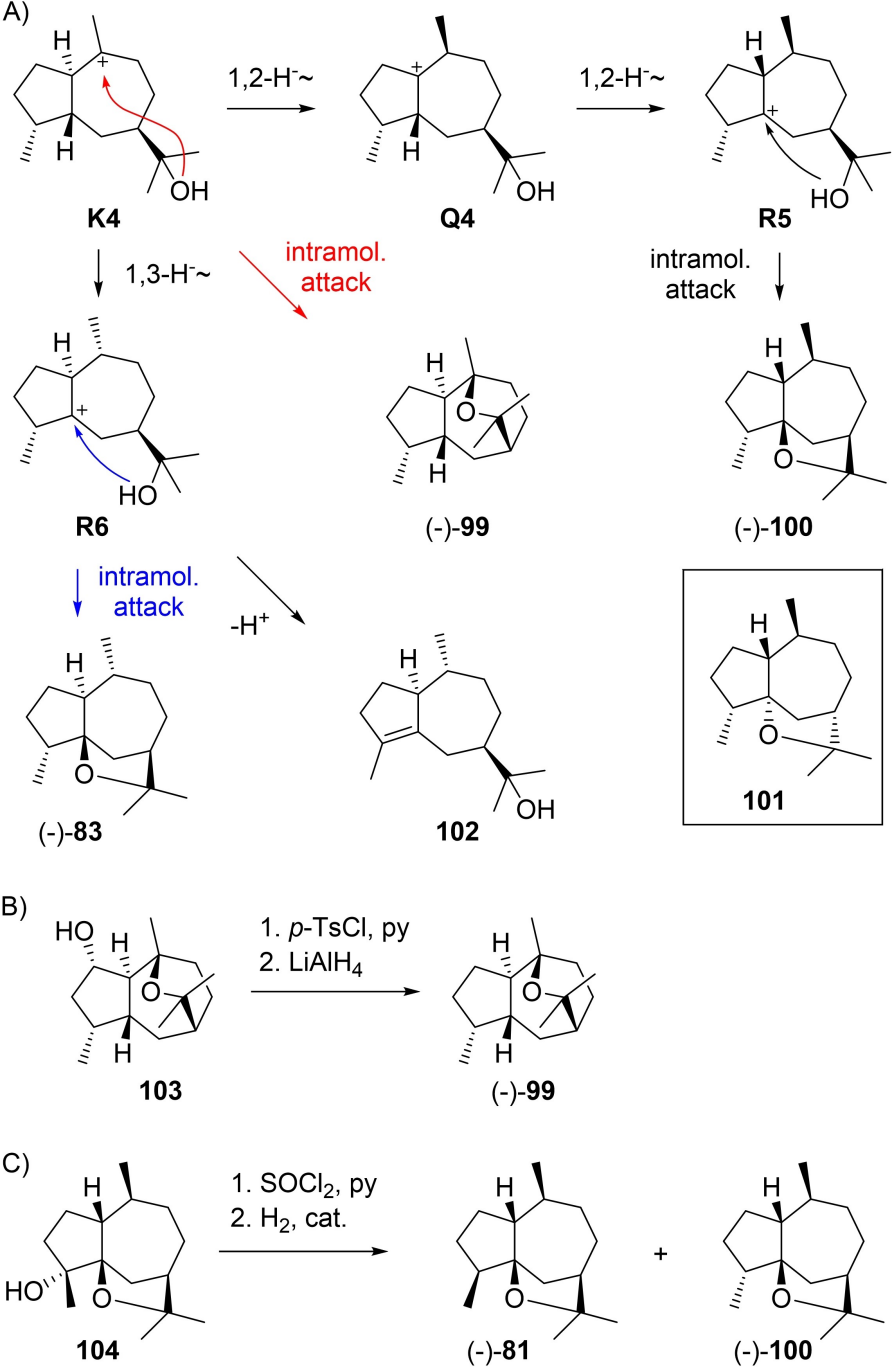
A) Guaiols derived from **K4**. B) Correlation of **103** with **99**. C) Correlation of **104** with **81** and **100**.

Guiaols from **K4** are given in Scheme 27A. A direct ring closure explains the formation of (−)‐kessane (**99**) that is known from the roots of several Japanese *Valeriana* species (kesso, [α]_D_=−7.2).^[230]^ Its structure including absolute configuration was established by correlation with known α‐kessyl alcohol (**103**)^[231]^ that was converted into **99** by tosylation and treatment with LiAlH_4_ (Scheme 27B),^[230]^ and by enantioselective synthesis from (+)‐aromadendrene.^[232]^ Kessane (**99**) was later isolated again from *Senecio*,[^233^, ^234^, ^235^] *Bothriochloa intermedia*,^[40]^ *Prostanthera ovalifolia*,^[179]^ *Olearia phlogopappa* ^[236]^ and *Machaerium multiflorum*.^[237]^ Two sequential 1,2‐hydride shifts via **Q4** to **R5** and ring closure give rise to (−)‐liguloxide (**100**) from *Ligularia* ([α]_D_=−52.8).^[228]^ Initially, the structure of **101** was assigned to this compound, but elimination of water from **104** and catalytic hydrogenation yielded guaioxide (**81**) and liguloxide (**100**), showing that these compounds must be C4 epimers (Scheme 27C).^[229]^ A 1,3‐hydride shift from **K4** to **R6** and deprotonation lead to **102** that is observed in guaiacwood oil,^[114]^ while intramolecular attack of the alcohol to the cation in **R6** offers an explanation for the biosynthesis of **83** from *Ligularia* (Scheme 27A).^[202]^

### 4.6 Cyclisation of hedycaryol by protonation at C10

**Scheme 28:**
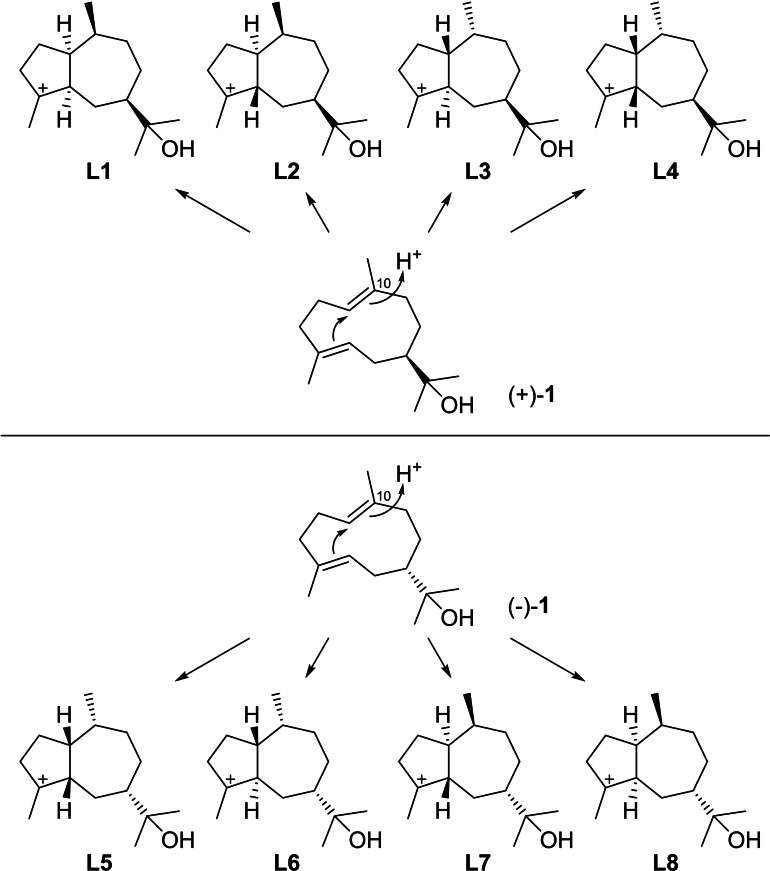
Cyclisation reactions of **1** induced by reprotonation at C10 towards intermediates **L1**–**L8**.

The cyclisation of hedycaryol can also be initiated by protonation at C10 (Scheme 28), leading to the two enantiomeric series of cationic intermediates **L1**–**L4** from (+)‐**1** and **L5**–**L8** from (−)‐**1**. Again, no examples of natural products for the series from (−)‐**1** with unambiguously determined absolute configuration are available, and thus the further discussion will be limited to the compounds derived from (+)‐**1**.

**Scheme 29:**
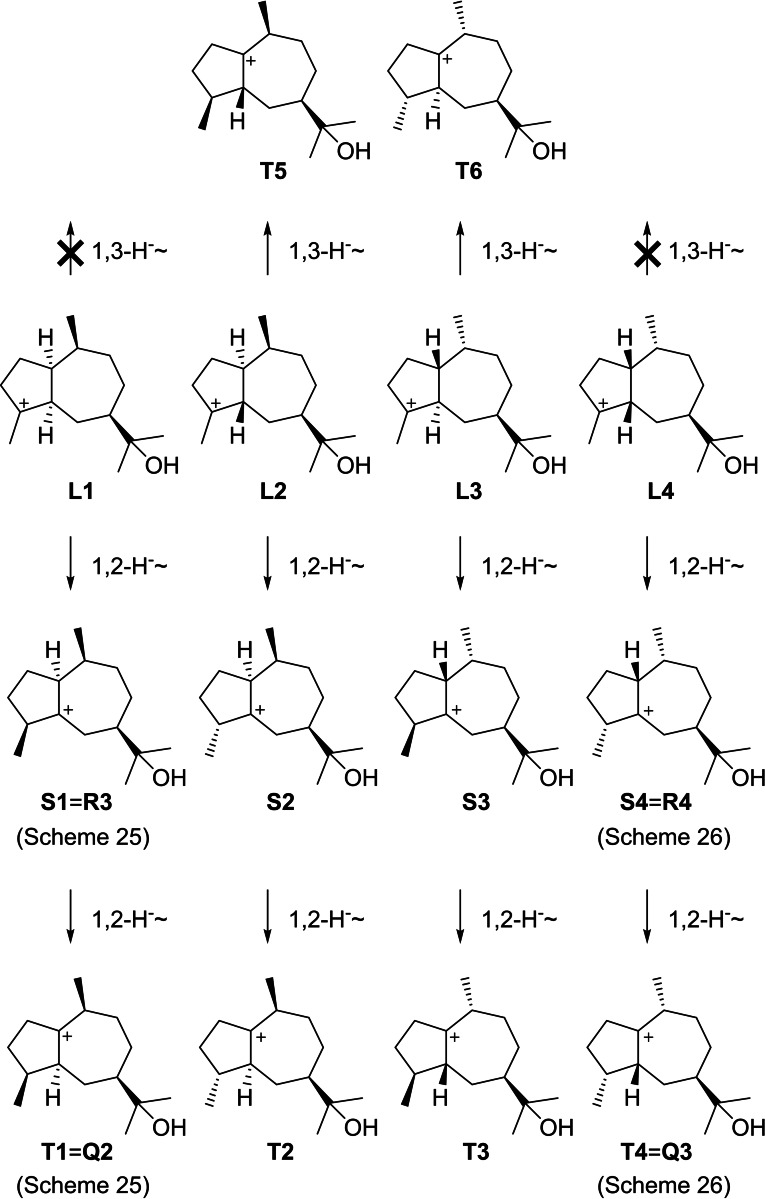
Downstream steps from **L1**–**L4** by 1,2‐ and 1,3‐hydride migrations.

It is interesting to note that subsequent hydride transfers in some cases lead to the same intermediates as discussed above (Scheme 29). Specifically, 1,2‐hydride migrations from **L1**–**L4** result in **S1** ‐ **S4** and then **T1**–**T4**. Herein, **S1** and **T1** are equal to **R3** and **Q2** (Scheme 25), while **S4** and **T4** are equal to **R4** and **Q3**, respectively (Scheme 26). Compounds that were already discussed above and could have an alternative biosynthesis along these lines will not be presented here again. Furthermore, **L2** and **L3** can react in 1,3‐hydride migrations to **T5** and **T6**, respectively. Analogous steps are sterically not possible for **L1** and **L4**, as was also shown by DFT calculations.^[18]^

### 4.7 Guaiols potentially arising from hedycaryol by C10 protonation

**Scheme 30:**
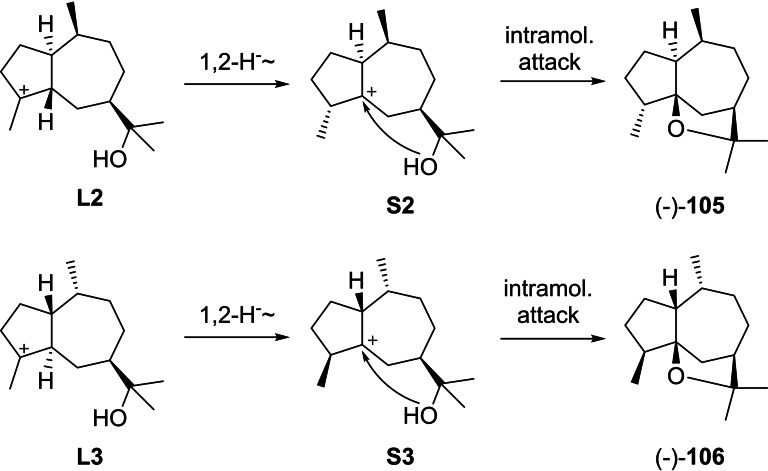
Compounds **105** and **106** that may arise by C10 protonation of **1**.

Notably, most bicyclic 5–7 membered compounds from (+)‐**1** can be rationalised through a cyclisation induced by protonation at C4. While the biosynthesis in many cases has not been studied in detail and it is often unknown, whether compounds are formed from (+)‐**1** by C4 or C10 protonation, only two more compounds exist whose biosynthesis cannot be easily understood by C4 protonation (Scheme 30). In these cases C10 protonation could more reasonably explain their direct biosynthesis, which could lead to the only two remaining compounds (−)‐1‐*epi*‐liguloxide (**105**) and (−)‐bulnesoxide (**106**) that will be discussed here.

Starting from **L2**, a 1,2‐hydride shift to **S2** and intramolecular attack of the alcohol can give rise to (−)‐**105** ([α]_D_=−25.6),^[238]^ while similar reactions from **L3** via **S3** can lead to (−)‐**106** ([α]_D_=−8.2).^[239]^ In fact, both compounds were so far only obtained by synthesis,[^238^, ^239^] which questions whether a protonation of (+)‐**1** at C10 in a terpene synthase catalysed reaction is relevant for any natural product, as it seems that the formation of all compounds that were isolated from natural sources can be explained through cyclisation of (+)‐**1** by C4 protonation and the subsequent reactions discussed above.

## 5 Conclusions

Many natural products are known that biosynthetically arise from hedycaryol (**1**). Plants generally make the compounds derived from (+)‐**1**, while bacteria and fungi produce compounds derived from (−)‐**1**, and because significantly more research has been done on plants than on bacteria and fungi, most known compounds originate from (+)‐**1** and thus have 7*R* configuration. For many compounds, the absolute configurations have been secured by chemical correlations including total synthesis, but sometimes the situation is not fully resolved or even confusing. Particularly the assignments of optical rotations can be erroneous, which can easily happen if impure materials have been measured and the minor contaminants may have large optical rotations of opposite sign in comparison to the investigated compound. Especially the cases of the enantiomers 5‐*epi*‐10‐*epi*‐γ‐eudesmol and 7‐*epi*‐γ‐eudesmol that were both synthesised from the enantiomers of dihydrocarvone,[^131^, ^187^] but then both reported to have negative optical rotations, and eventually of α‐eudesmol for which the old work consistently reported a positive optical rotation, while new data support a negative value, deserve a revision.

Cyclisations of hedycaryol can either give a 6–6 membered bicyclic system, which represents the majority of cases. These cyclisations are always induced by protonation at C1, leading to a tertiary cationic intermediate, and not at C4 that would give a less stable and disfavoured secondary cation. Alternatively, a 5–7 membered bicyclic system can be formed for which protonations of **1** at C4 or C10 could potentially be relevant. As we demonstrated here, all compounds can be explained through protonation at C4, with only two remaining cases whose biosynthesis would need C10 protonation, but these compounds are only known as synthetic materials. Therefore, it seems that C4 protonation may serve as the general mechanistic model towards 5–7 bicyclic compounds, and we argue that this is because protonations at the C1=C10 double bond may preferentially happen at C1 to result in the 6–6 membered bicyclic systems. This reflects the situation that we have recently summarised for compounds derived from germacrene A for which the analysis of all known compounds also suggested that protonations of the C1=C10 double bond preferentially happen at C1 with formation of 6–6 membered bicyclic compounds, while protonations at the opposite C4=C5 double bond are directed toward C4 and induce formation of 5–7 membered bicyclic sesquiterpenes.^[17]^ Taken together, hedycaryol and germacrene A show – not surprisingly – the same intrinsic reactivity, and the question of forming a 6–6 versus a 5–7 bicyclic ring system is a question of which of the two double bonds in the macrocycle becomes reprotonated. Notably, for patchoulol synthase different mechanisms with C4 and C10 protonation of germacrene A were discussed in the literature,[^240^, ^241^, ^242^] and a recent mechanistic study from our laboratories has shown that C4 protonation is relevant for this molecule.^[243]^ However, clearly more research is required to further confirm the general hypothesis outlined here, because for most compounds the biosynthesis has not been studied experimentally.

## Conflict of interest

The authors declare no conflict of interest.

6

## Biographical Information

*Jeroen S. Dickschat studied Chemistry at TU Braunschweig and completed his PhD in 2004. He then moved for postdoctoral stays to Saarland University and the University of Cambridge. In 2008, he became a group leader at TU Braunschweig. In 2014, he was appointed Professor of Organic Chemistry and Biochemistry at the University of Bonn. His research interests span the synthesis and biosynthesis of natural products*.

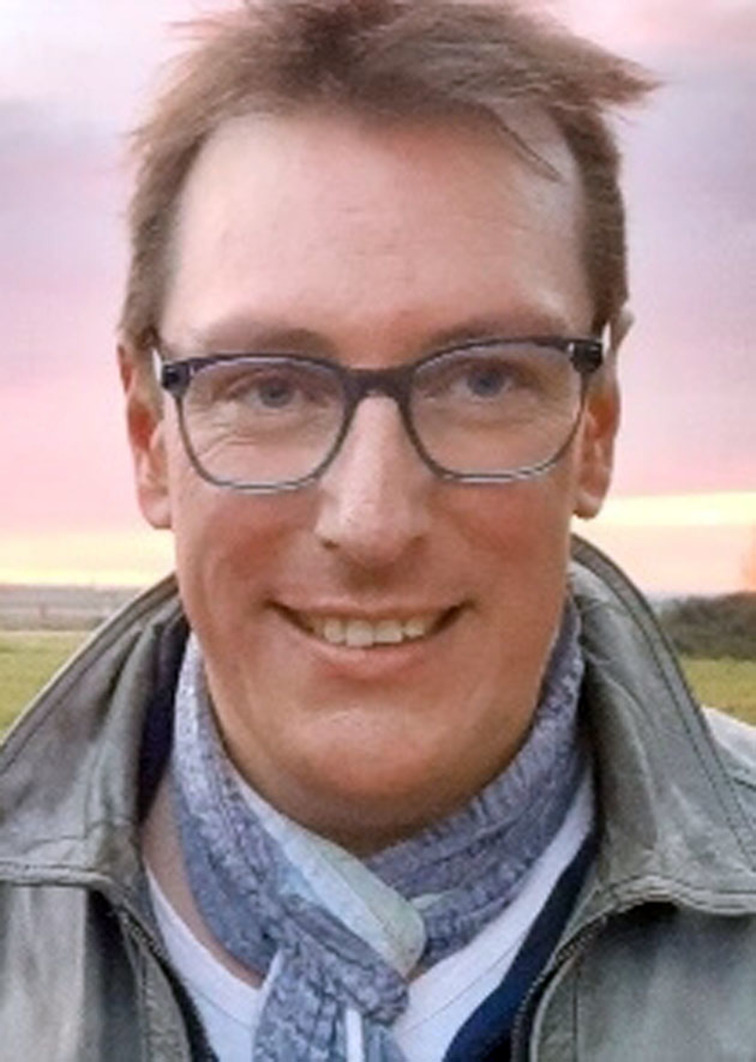

## Biographical Information

*Houchao Xu graduated from China Pharmaceutical University with a B.Sc. degree in 2015. He then obtained his M.Sc. degree from Kunming Institute of Botany, Chinese Academy of Sciences. In September 2019, he started his doctoral study in the group of Prof. Dickschat at the University of Bonn. His research focuses on the chemical synthesis and biosynthesis of terpenes and polyketides*.

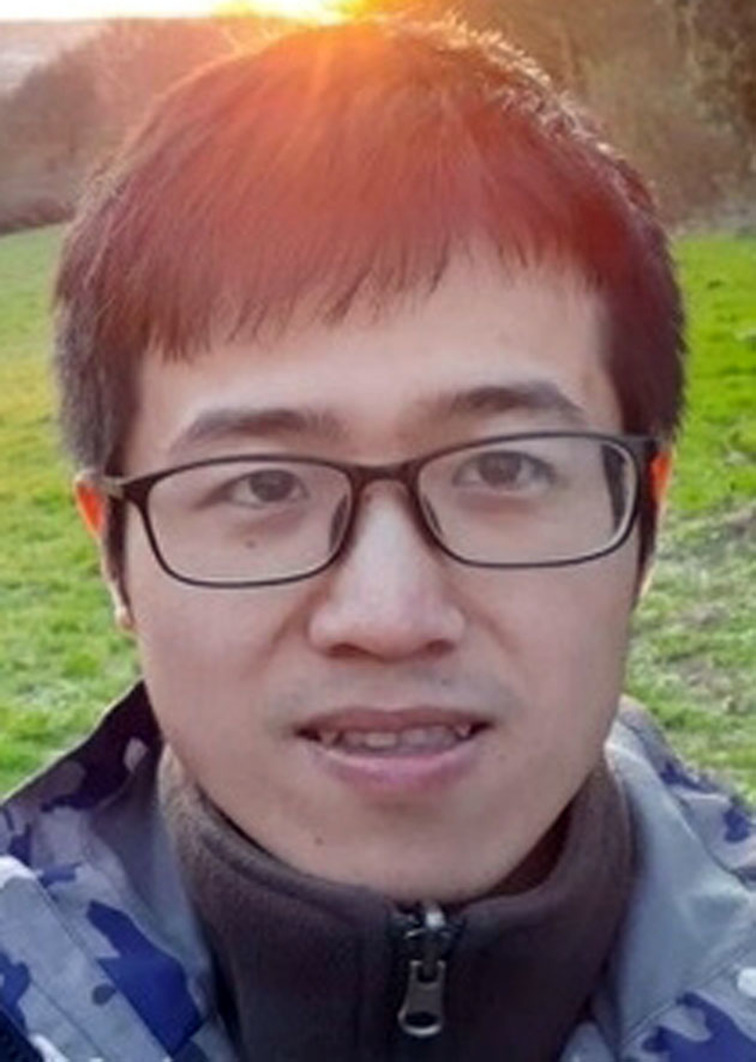

## Data Availability

Data sharing is not applicable to this article as no new data were created or analyzed in this study.
